# Engineering Synergistic Intra‐/Intermolecular Conformational Locking via Side‐Chain Branching: A β‐Sheet‐Inspired Strategy for Planar NIR‐II Phototheranostic Aggregates

**DOI:** 10.1002/advs.76688

**Published:** 2026-07-24

**Authors:** Cheng Lu, Qian Jia, Bo Wu, Chao Li, Ryan T. K. Kwok, Tie‐Gen Chen, Jianwei Sun, Zheng Zhao, Teng‐Teng Chen, Zhongliang Wang, Jacky W. Y. Lam, Ben Zhong Tang

**Affiliations:** ^1^ Department of Chemistry Hong Kong Branch of Chinese National Engineering Research Center for Tissue Restoration and Reconstruction Division of Life Science and State Key Laboratory of Molecular Neuroscience The Hong Kong University of Science and Technology Kowloon Hong Kong China; ^2^ Lab of Molecular Imaging and Translational Medicine (MITM) Engineering Research Center of Molecular and Neuro Imaging Ministry of Education School of Life Science and Technology Xidian University & International Joint Research Center for Advanced Medical Imaging and Intelligent Diagnosis and Treatment Xi'an Shaanxi China; ^3^ School of Environmental and Chemical Engineering Wuyi University Jiangmen Guangdong China; ^4^ Department of Chemistry The Hong Kong University of Science and Technology Kowloon Hong Kong China; ^5^ Zhongshan Institute for Drug Discovery Shanghai Institute of Materia Medica Chinese Academy of Sciences Zhongshan China; ^6^ Guangdong Basic Research Center of Excellence for Aggregate Science School of Science and Engineering The Chinese University of Hong Kong (Shenzhen) Shenzhen Guangdong China

**Keywords:** aggregate‐state engineering, bio‐inspired strategy, NIR‐II phototheranostics, noncovalent conformational locking, side‐chain branching

## Abstract

Engineering stable noncovalent conformational locks (NoCL) network at the aggregate level is essential for developing phototheranostic aggregates (PTAs) with enhanced rigidity and light‐harvesting ability, but remains a challenge due to complex intermolecular interactions. Inspired by β‐sheet proteins, where branched side chains act as steric directors to program backbones into interlocked architectures, a side‐chain isomerization strategy is proposed to manipulate analogous NoCL networks for constructing planar‐structured NIR‐II multimodal PTAs. Two isomeric pairs with linear (*l*‐series) and branched (*b*‐series) alkyl side‐chains are synthesized. Crystallographic analysis reveals that the *b*‐series, driven by directional S···O/F interactions, adopts a fully planar conformation stabilized by a synergistic dual NoCL network, whereas *l*‐series exhibits twisted conformations. Theoretical calculation and femtosecond transient absorption spectra confirm the dual NoCL network effectively narrows the energy gap and optimizes excited‐state energy dissipation pathway, resulting in comprehensive enhancement in phototheranostic performance including superior ROS generation, higher NIR‐II brightness and excellent photothermal properties compared to *l*‐series NPs. Notably, the exceptional performance of *b*‐3CPFIC NPs enables it to serve as an ideal candidate in multimodal NIR‐II phototheranostics of tumors. This work establishes a novel design paradigm for multifunctional PTAs and elucidates aggregate‐level structure–property relationships.

## Graphical abstract

A bio‐inspired side‐chain branching strategy is developed to orchestrate a dual noncovalent conformational locking (NoCL) network, enabling the construction of perfectly planar NIR‐II phototheranostic aggregates. Such structural precision maximizes light‐harvesting efficiency and optimizes exciton dynamics, ensuring synchronized enhancements in NIR‐II brightness, ROS generation, and photothermal conversion, providing a novel paradigm for designing high‐performance “one‐for‐all” phototheranostics.

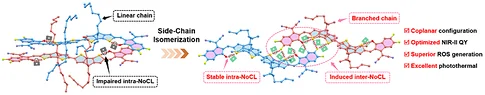

## 1 Introduction

The convergence of diagnostic imaging and light‐activated therapy within the second near‐infrared (NIR‐II) biological window has emerged as a paradigm in precision medicine, owing to its deep tissue penetration, high spatiotemporal resolution, and reduced off‐target effects [1, 2, 3, 4]. In particular, “all‐in‐one” multimodal platforms that synergistically integrate NIR‐II fluorescence imaging, photoacoustic (PA) imaging, photodynamic therapy (PDT), and photothermal therapy (PTT) are highly attractive for providing complementary diagnostic data and therapeutic outcomes [5, 6, 7, 8, 9, 10]. However, developing a single molecular platform that efficiently supports all these modalities remains a formidable challenge, primarily due to intrinsic photophysical constraints such as competing energy dissipation pathways and limited light absorption, which often force performance trade‐offs [11, 12, 13, 14]. To overcome these bottlenecks, extensive efforts have focused on molecular‐level engineering, aiming to enhance light‐harvesting ability by extending π‐conjugation or rigidifying the molecular backbone [15, 16, 17, 18, 19]. Introducing intramolecular noncovalent conformational locks (NoCLs) has proven effective in improving molecular planarity and rigidity [20, 21, 22, 23, 24, 25]. Nevertheless, such single‐molecule optimization strategy inherently overlooks the fact that phototheranostic agents function in vivo predominantly as aggregates. In the aggregate state, uncontrolled intermolecular interactions can distort the pre‐optimized molecular conformation and deteriorate multimodal performance. This mismatch between single‐molecule design and aggregate‐state operation underscores the urgent need for aggregate‐level design principles that explicitly consider molecular packing, intermolecular interactions, and conformational behavior [26, 27, 28, 29, 30].

Within this framework, constructing robust intermolecular NoCLs becomes critical. They can further stabilize the aggregated structure, enhance backbone planarity, and suppress vibrational/rotational energy loss, thereby maximizing light harvesting and steering excited‐state energy toward desired outputs [31, 32]. Nevertheless, the design of intermolecular NoCLs is inherently challenging due to the difficulty in predicting and precisely controlling intermolecular interactions, and their stability remains a concern owing to the weak nature of noncovalent forces [33, 34, 35, 36]. Therefore, a key challenge is to develop a cooperative strategy that can simultaneously stabilize intramolecular NoCLs and induce well‐defined intermolecular NoCLs, and to establish the fundamental structure–performance relationships at the aggregate level [37].

In addressing this challenge, we draw inspiration from nature's sophisticated assembly blueprints. The β‐sheet motif in proteins serves as an ideal model for programming synergistic conformational locks [38, 39]. In β‐sheets, a directional hydrogen‐bonding network between peptide backbones provides the primary driving force for assembly, while the distinct topology of β‐branched side chains acts as a decisive steric director. These branched side chains program the assembly pathway by exerting precise steric pressure, effectively locking adjacent strands into a registered, interlocked topology [40, 41]. This mechanism suggests that coupling an interaction‐active backbone (featuring S, O or F sites) with programmable side‐chain topology can allow these side chains to serve as conformational templates, cooperatively orchestrating both intra‐ and intermolecular NoCLs to anchor the molecular skeleton into an absolute planar aggregate state that significantly boosts phototheranostic performance [42, 43].

Herein, we propose a side‐chain isomer‐guided NoCL network strategy that employs branched side chains as conformational directors to synchronously modulate intra‐ and intermolecular NoCLs. This enables the construction of high‐performance NIR‐II OPTAs with a fully coplanar architecture (Figure 1A). As a representative model system, the CP‐FIC framework was synthesized with either linear (*l*‐series) or branched (*b*‐series) alkyl chains. To evaluate the robustness of this principle across diverse electronic environments, the donor core was systematically extended from 2CP to 3CP (Figure 1B). Strikingly, while maintaining similar single‐molecule photophysics, all *b*‐series nanoparticles (NPs) exhibited comprehensively enhanced performance, including superior light‐harvesting, reactive oxygen species generation, NIR‐II fluorescence brightness, and photothermal conversion. These improvements effectively overcome the traditional photophysical trade‐offs observed in their *l*‐series counterparts. Single‐crystal analysis and theoretical calculations revealed that the branched derivative adopts a perfectly coplanar conformation stabilized by a cooperative dual NoCL network, where the optimal steric hindrance from branched side chains suppresses detrimental π–π stacking interference (Figure 1C). In contrast, the linear analogue displays a twisted skeleton due to disrupted intramolecular locking. Furthermore, femtosecond transient absorption (fs‐TA) spectroscopy demonstrated that the dual NoCL network promotes favorable nonradiative relaxation and prolongs the triplet exciton lifetime, collectively contributing to the superior phototheranostic properties (Figure 1D). The optimal *b*‐3CPFIC NPs were successfully applied for NIR‐II fluorescence/photoacoustic imaging‐guided synergistic PDT/PTT (Figure 1E). This work establishes a novel paradigm for designing efficient all‐in‐one phototheranostic platforms and provides fundamental insights into conformation‐dependent performance at the aggregate level.

**FIGURE 1:**
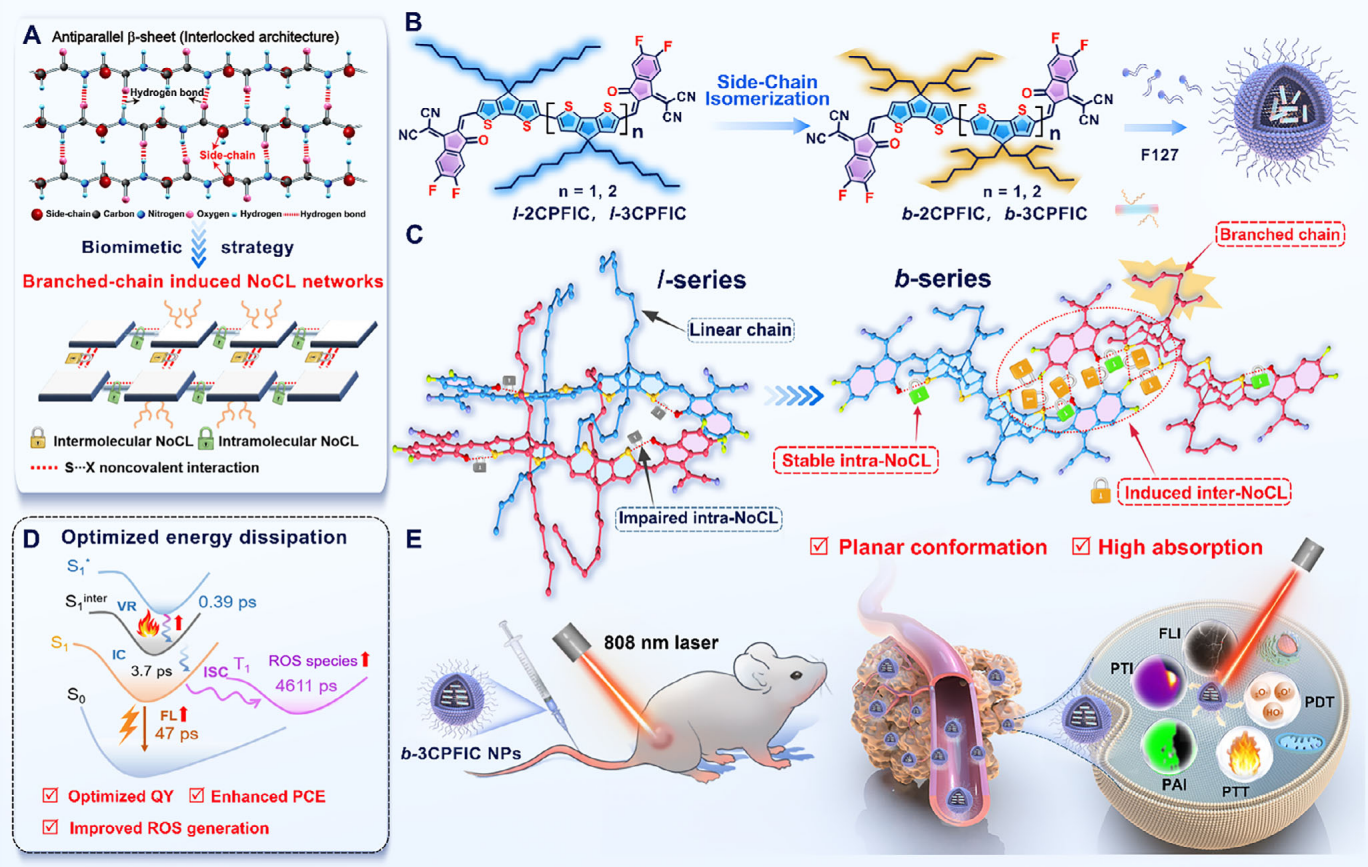
(A) Schematic of a β‐sheet‐inspired branched‐chain regulation strategy for intra‐/intermolecular NoCL networks. (B) Illustration of nonfused‐ring A‐D‐A‐structured molecules with linear alkyl side‐chain and branched alkyl side‐chain groups, *l*‐2CPFIC, *b*‐2CPFIC, *l*‐3CPFIC and *b*‐3CPFIC, and fabrication to the corresponding nanoparticles. (C) The alkyl side‐chain topology isomerization manipulated intra‐/intermolecular NoCL which facilitated the molecular planarity and locked packing in the aggregated state. (D) The optimized energy dissipation pathway of *b*‐series NPs. (E) The NIR‐II fluorescence imaging‐guided multifunctional phototherapy of *b*‐3CPFIC NPs.

## 2 Results and Discussion

### 2.1 Molecular Design and Photophysical Properties

To translate the bio‐inspired side‐chain steering principle into a functional aggregate system, four nonfused‐ring acceptor–donor–acceptor (A–D–A) structured conjugated molecules with different alkyl configurations of linear alkyl chain (*l*‐2CPFIC and *l*‐3CPFIC) or branched alkyl chain (*b*‐2CPFIC and *b*‐3CPFIC) were designed and synthesized. In these compounds, the 4H‐cyclopenta[1,2‐b:5,4‐b′]dithiophene (CP) donor and the electron‐deficient fluorinated 2‐(3‐oxo‐3‐dihydroinden‐1‐ylidene)malononitrile (FIC) acceptor were strategically employed to construct an interaction‐active backbone. Their inherent capacity to form multifaceted S⋯O and S⋯F non‐covalent interactions serves as the fundamental prerequisite for establishing the dual NoCL network. Further considering both synthetic accessibility and the potential impact of chain length on NoCL formation, octyl and 2‐ethylhexyl were selected as the corresponding alkyl chain isomers [44]. Among them, the β‐branched 2‐ethylhexyl group provides a precise steric balance, utilizing its one‐carbon spacer to preserve the planar intramolecular lock and its bulky branch to restrict parallel π‐π stacking, thereby enforcing an end‐to‐end intermolecular locking alignment. To verify the strategy's generality across diverse electronic environments, the donor core was extended from 2CP to 3CP, providing a systematic test to evaluate whether the side‐chain‐steered NoCL network remains robust against increased conjugation and structural perturbations. These target molecules were obtained from aldehyde intermediates and FIC via Knoevenagel condensation in high yields (Scheme S1). More detailed synthetic procedures and structure characterization (^1^H and ^13^C NMR spectroscopy and high‐resolution mass spectrometry (HRMS)) were described in Figures S1S11. The as synthesized molecules showed excellent solubility in common organic solvents.

The photophysical properties of *l*‐2CPFIC, *b*‐2CPFIC, *l*‐3CPFIC and *b*‐3CPFIC in mono‐dispersed state were investigated first. Driven by the identical conjugated backbone within each pair, the *l*‐ and *b*‐series exhibited generally similar absorption and emission profiles in THF (Figure S12). Their consistent spectral responses to varying solvent polarities indicated negligible differences in intramolecular charge transfer (TICT) properties (Figure S13). Nevertheless, a slight absorption blue‐shift was consistently observed in the *b*‐series, which arises from backbone twisting induced by localized steric hindrance and the resultant microenvironment shielding effects (Figure S14). Beyond the side‐chain variations, compared to their 2CP‐based counterparts, 3CP‐based molecules exhibited both red‐shifted absorption and emission, which are consistent with the narrower energy band gap resulting from the elongated π‐conjugation (Figure S15).

To endow these hydrophobic compounds with good water solubility and biocompatibility, *l*‐2CPFIC, *b*‐2CPFIC, *l*‐3CPFIC and *b*‐3CPFIC were fabricated into NPs through the ultrasound‐assisted nanoprecipitation method by using an amphiphilic copolymer F127 as encapsulation matrix. Results from dynamic light scattering showed that the average hydrodynamic diameters of *l*‐2CPFIC NPs, *b*‐2CPFIC NPs, *l*‐3CPFIC NPs and *b*‐3CPFIC NPs were 103.3, 68.3, 85.6, and 64.6 nm, respectively (Figure 2A), which were suitable for the passive target accumulation of tumor through the enhanced permeability and retention (EPR) effect. Notably, the *b*‐series NPs were consistently smaller than their *l*‐series counterparts, a trend substantiated by our packing density calculations (Table S1), which indicated that the branched side chains promoted a more compact molecular arrangement. The *b*‐3CPFIC NPs showed a uniform spherical morphology and a moderately negative zeta potential. Their nearly unchanged size in phosphate‐buffered saline (PBS) and PBS + 10% fetal bovine serum over 7 d confirmed good colloidal stability (Figure S16).

**FIGURE 2:**
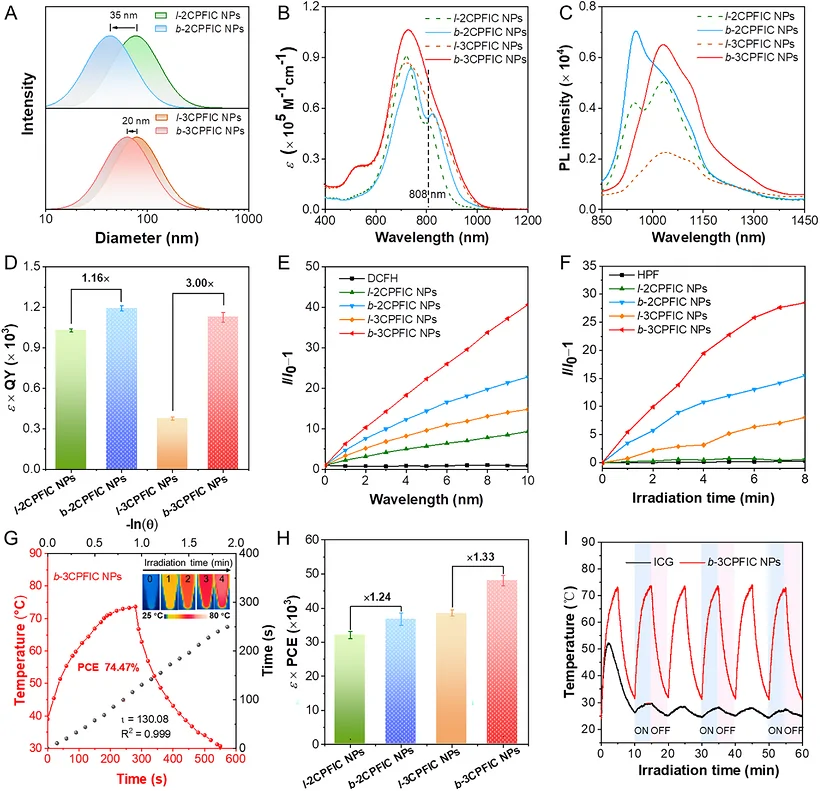
(A) The size distribution of NPs in water measured by DLS. (B) Absorption spectra and (C) PL spectra of *l*‐2CPFIC NPs, *b*‐2CPFIC NPs, *l*‐3CPFIC NPs and *b*‐3CPFIC NPs (10 µm) in water. (D) The brightness (*ε* × QY) of four NPs. (E) Total ROS generation and •OH generation efficiency of four NPs (10 µm) upon laser irradiation (808 nm, 0.5 W cm^−2^) using DCFH‐DA and HPF as indicator, respectively. (G) Photothermal conversion efficiency of *b*‐3CPFIC NPs. The inset shows the photothermal images of the NPs under 808 nm laser irradiation. (H) The photo‐thermal conversion performance (*ε* × PCE) of four NPs upon 808 nm laser irradiation (0.5 W cm^−2^). (I) Thermostability of *b*‐3CPFIC NPs and ICG in water under 808 nm radiation (0.5 W cm^−2^) for six cycles.

To gain further insight into the effect of alkyl chain on photophysical properties of aggregated state, their UV‐vis‐NIR and photoluminescence (PL) spectroscopies were then studied. As shown in Figure 2B, the *b*‐series NPs exhibited red‐shifted absorption and higher molar extinction coefficients (*ε*) at 808 nm compared with their *l*‐series counterparts. In contrast to the slight blue‐shift observed for the *b*‐series in dilute solution, this aggregate‐state red‐shift indicated that branched side chains promoted extended effective conjugation after NP formation, thereby enlarging the absorption reservoir. Upon 808 nm laser irradiation, these NPs emitted bright NIR‐II emission extended to 1400 nm (Figure 2C). Notably, the *b*‐series NPs exhibited higher NIR‐II brightness (*ε* × QY) compared to the *l*‐series counterparts. In particular, *b*‐3CPFIC NPs showed significant enhancement of fluorescence brightness, almost 3.5 folds higher than that of *l*‐3CPFIC NPs (Figure 2D, S17 and S18). These results indicated that side‐chain isomerization had a significant impact on the optical performance of NPs in the aggregate level. Given the potential influence of branch‐chain chirality on photophysical properties, we systematically performed circular dichroism (CD) spectroscopic measurements (Figure S19). The absence of detectable CD signals of *b*‐2CPFIC NPs and NPs *b*‐3CPFIC NPs conclusively demonstrated that these optical performance originates from achromatic configuration rather than stereochemical induction. Furthermore, the ROS generation capacity and photothermal characteristics of these NPs were investigated in aqueous medium. DCFH‐DA was used as the indicator to monitor the total ROS generation efficiency. As the 808 nm laser continuously irradiated the PBS solutions containing respective *l*‐2CPFIC NPs, *b*‐2CPFIC NPs, *l*‐3CPFIC NPs and *b*‐3CPFIC NPs, the fluorescence intensity of DCFH‐DA at 525 nm was remarkably boosted, which reached about 12‐fold, 20‐fold, 8‐fold and 40‐fold, respectively, compared with the original emission intensity without light irradiation (Figures 2E and S20). Subsequent investigation into the specific ROS types indicated that these NPs efficiently generated •OH and •O_2–_ through a type‐I proces**s**. This was initially suggested by the minimal ABDA absorption changes alongside the significantly increased fluorescence of HPF and DHR123 over time (Figures 2F and S21–S23), and further validated by electron paramagnetic resonance (EPR) spectra showing prominent DMPO‐•OH signals with negligible TEMPO triplet signals (Figure S36). Furthermore, as another important parameter of phototheranostic properties, the photo‐induced heat generation capacity of these NPs was investigated. Compared to the *l*‐series NPs, the *b*‐series NPs showed higher photothermal conversion efficiencies (PCE), with *b*‐3CPFIC NPs reaching a PCE as high as 74.47% (Figures 2G and S25). Considering that the final heating effect also depends on the absorption capacity and PCE of the NPs, their actual photothermal effect was evaluated by *ε* × PCE. As shown in Figure 2H, *b*‐2CPFIC and *b*‐3CPFIC NPs displayed better heat‐generating performance than *l*‐series counterparts, which may be attributed to increased number of surface‐exposed molecules on the NPs for boosting motion. Meanwhile, 3CP‐based NPs exhibited superior photothermal conversion performance over 2CP‐based NPs, likely due to their twisted conformations enabling more space for molecular motion promoting non‐radiative decay (Figure S26). These data firmly indicated that brightness, ROS generation and photothermal conversion performance in the aggregate state, which were generally considered as contradictions, were efficiently enhanced simultaneously via the alkyl side‐chain topology isomerization strategy.

Moreover, the photothermal stability of *b*‐3CPFIC NPs was appraised by observing the temperature change in six continuous off/on cycles upon 808 nm laser irradiation. Compared with a commercial dye called indocyanine green (ICG), the aqueous solution sample of *b*‐3CPFIC NPs exhibited a steady‐state temperature alteration with the maximized elevated temperature showing tiny change during the heating and cooling cycle, indicating the outstanding photothermal stability (Figure 2I). In addition, the temperature elevation of *b*‐3CPFIC NPs was proportionally dependent on the concentration as well as the laser power density, revealing the controllability of light‐to‐heat conversion (Figure S27). Notably, the temperature of *b*‐3CPFIC NPs could easily raise up to 55°C even at a low concentration of 5 µm, demonstrating the distinguished photothermal conversion performance. Crucially, besides this excellent photothermal robustness, *b*‐3CPFIC NPs also possessed exceptional photochemical stability. This was validated by tracking their UV–Vis absorption spectra under continuous laser exposure, where the negligible absorbance alteration over time confirmed their robust resistance to photobleaching (Figure S28). The above results showed that the ultra‐stable, high‐performance *b*‐3CPFIC NPs were promising for application in the field of multi‐modal phototheranostics.

### 2.2 The Excited‐State Dynamic Study

To elucidate the superior photophysical properties of *b*‐series NPs relative to *l*‐series counterparts, fs‐TA spectra upon 720 nm excitation were performed. Taking the *l*‐2CPFIC and *b*‐2CPFIC NPs as representative systems (Figure 3A, B), both samples exhibited strong ground state bleaching (GSB) in the 600–800 nm region, accompanied by excited‐state absorption peaks at 1017 nm and 1195 nm for *l*‐2CPFIC NPs, and at1003 and 1160 nm for *b*‐2CPFIC NPs (Figure 3B, E). The long‐lived ESA signal at approximately 1200 nm arises from intermolecular charge delocalization within nanoaggregates, as evidenced by the singular ESA band observed in THF‐dispersed single‐molecule state (Figure S29). Particularly, the prolonged ESA lifetime in *b*‐2CPFIC NPs demonstrated significantly strengthened intermolecular interactions that promote more efficient interchromophore charge transfer than *l*‐2CPFIC NPs (Figure S30). Spectral overlap between the emission profile (orange dashed line) and ESA bands necessitated advanced kinetic analysis through global lifetime analysis (GLA), thus a sequential four‐compartment model was employed to obtain best fitting (Figure 3C, F and Figure S31). Since the first and second species have similar evolution‐associated spectra (EAS), which can be assigned to hot singlet excited state (S_1_*) and the lowest singlet excited state (S_1_), and the S_1_* transforms into S_1_ via fast structural relaxation, with similar lifetime of 0.41 and 3.2 ps for *l*‐2CPFIC NPs, and 0.39 and 3.7 ps for *b*‐2CPFIC NPs. Notably, up to 81% of the excited population of *b*‐2CPFIC NPs underwent rapid non‐radiative depletion versus 80.4% in *l*‐2CPFIC NPs, confirming dominant non‐radiative decay pathway. This slightly higher non‐radiative fraction, combined with a larger intramolecular reorganization energy of *b*‐2CPFIC (7.65 kJ mol^−1^)) than *l*‐2CPFIC (6.62 kJ mol^−1^), jointly drives the superior PCE of *b*‐2CPFIC NPs (Figure 3G and Table S2). Furthermore, the fluorescence lifetime recorded by TCSPC (Figure S32) is 0.57 and 0.65 ns for *l*‐2CPFIC and *b*‐2CPFIC aggregates, respectively, are consistent with the lifetime scale of third species. Crucially, these sub‐nanosecond lifetimes suggested that the observed fluorescence predominantly originates from delocalized excitonic states. The longer lifetime in *b*‐2CPFIC NPs implied enhanced conformation rigidity compared to the linear counterpart after aggregation. More importantly, considering that the triplet excited states have longer lifetime than single excited states, it was reasonable to assign the four species in EAS to the triplet excited states via intersystem crossing (ISC) from delocalized singlet exciton. The obviously extended triplet lifetime in *b*‐2CPFIC NPs (4611 vs 1555 ps for *l*‐2CPFIC NPs) confirmed the boosted ISC process, directly correlating with the improved ROS generation (Figure 3H, I). This mechanistic framework was further validated in *l*‐3CPFIC/*b*‐3CPFIC NPs systems through analogous four‐compartment kinetic modeling (Figure S33). The more pronounced disparities in non‐radiative components and distinct triplet‐state lifetimes between *b*‐3CPFIC and *l*‐3CPFIC NPs collectively drive significantly enhanced PCE and ROS production capabilities observed in *b*‐series NPs (Figure S34).

**FIGURE 3:**
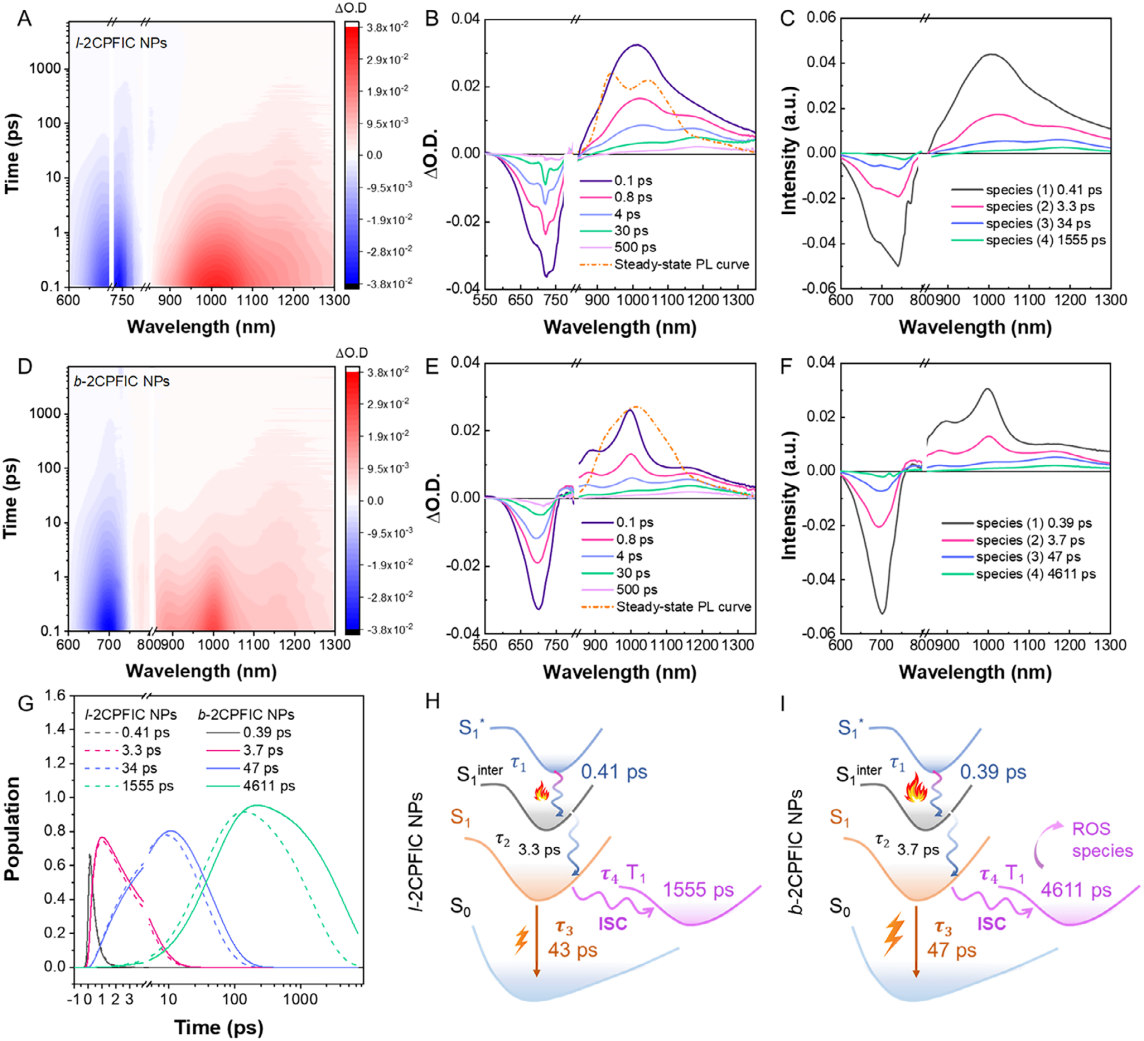
Raw fs‐TAS data and global analysis upon 720 nm excitation. 2D color fs‐TA mapping of (A) *l*‐2CPFIC NPs and (D) *b*‐2CPFIC NPs in water following photoexcitation with 720 nm laser plus. The fs‐TA spectra of (B) *l*‐2CPFIC NPs and (E) *b*‐2CPFIC NPs at different pump‐probe time delays. The corresponding evolution associated spectra (EAS) obtained from global lifetime analysis for (C) *l*‐2CPFIC NPs and (F) *b*‐2CPFIC NPs. (G) Time‐dependent amplitudes of individual species from GLA analysis for *l*‐2CPFIC NPs (dashed lines) and *b*‐2CPFIC NPs (solid lines). (H, I) Schematic illustration of excited‐state dynamic processes of *l*‐2CPFIC NPs and *b*‐2CPFIC NPs.

### 2.3 Single‐Crystal Analysis

The interesting phenomenon that *b*‐series NPs showed all optimized photophysical performances in comparison with *l*‐series NPs, which urged us to declare the reasons from the aggregate point of view. The single crystals of *l*‐2CPFIC (CCDC: 2404891) and *b*‐2CPFIC (CCDC: 2404889) with metallic luster were successfully obtained by slow diffusion at 4°C [45]. Detailed crystallographic data revealed S‐shaped conformations are stabilized by intramolecular NoCL via S···O interaction (Table S3). Interestingly, *b*‐2CPFIC displayed short S···O distance (2.65 Å) and a perfectly planar backbone with dihedral angle of 0° between CPs and between CPs and FICs, promoting enhanced absorption. In contrast, *l*‐2CPFIC exhibited longer S···O distance (2.74 Å) and more twisted molecular backbone with large angle between CPs (17.86°) and between CP and FIC (left: 12.86°, right: 9.55°) compared to *b*‐2CPFIC, aligning with the lower *ε* of *l*‐2CPFIC NPs (Figure 4A, B). This discrepancy might stem from variations in intermolecular non‐covalent interactions resulting from side‐chain isomerization. Consequently, a detailed investigation into the molecular packing mode of *b*‐2CPFIC and *l*‐2CPFIC was conducted. As predicted, the side‐chain isomers exhibited completely different stacking manners. In the *l*‐2CPFIC crystal, a unique face‐to‐face π‐core interaction between the CP unit in the adjacent molecules was observed, which may enhance the π–π interaction to quench the fluorescence emission (Figure 4C). Driven by the entanglement of flexible linear chains, this out‐of‐plane π–π pulling and the resulting alkyl‐backbone steric hindrance collectively disrupted the intramolecular lock to twist the backbone. In the same layer, the backbones were observed to be independent of each other, with no interactions noted due to the isolating effect of the side chains on the backbones. This was similar with packing for most traditional crystals with multiple side chains, which allowed for a loose stacking in the same layer and formed a honeycomb‐shaped network (Figure 4E). Unlike *l*‐2CPFIC, *b*‐2CPFIC exhibited attenuated CP‐to‐CP interactions with a large distance (7.04 Å) due to the robust steric shielding of the branched side chains. This spatial isolation significantly weakened the destructive out‐of‐plane π‐π stacking, which effectively prevented fluorescence quenching and spared the backbone from distorting strains to perfectly preserve its intramolecular planarity (Figure S35). Excitingly, guided by this in‐plane steric confinement, the molecular conjugated backbones were locked in an end‐to‐end way via intermolecular noncovalent interaction including S···F (2.903 Å/2.911 Å), C‐H···O (2.402 Å) (marked with red dotted lines in Figure 4D). This intermolecular NoCL could stabilize the conformation of the molecules and promote the planarity of the parallel molecules in the aggregate state, culminating in the formation of an absolutely planar 2D layer structure (Figure 4F). Furthermore, XRD analysis and molecular dynamics simulations revealed that *b*‐2CPFIC NPs maintains coplanar conformations and packing arrangements closely resembling those observed in its single‐crystal structure (Figures S36 and S37). Notably, the *b*‐2CPFIC NPs exhibit significantly enhanced structural ordering compared to their counterpart *l*‐2CPFIC NPs, which can be attributed to stronger directional intermolecular interactions such as S···F interaction. Overall, the side‐chain isomerization strategy effectively stabilized intramolecular NoCL and promoted intermolecular NoCL, resulting in a completely planar conformation within aggregates. This is beneficial for improving the absorptive capacity and simultaneously enhancing their fluorescence brightness, ROS efficiency and photothermal effect of NIR‐II PTAs.

**FIGURE 4:**
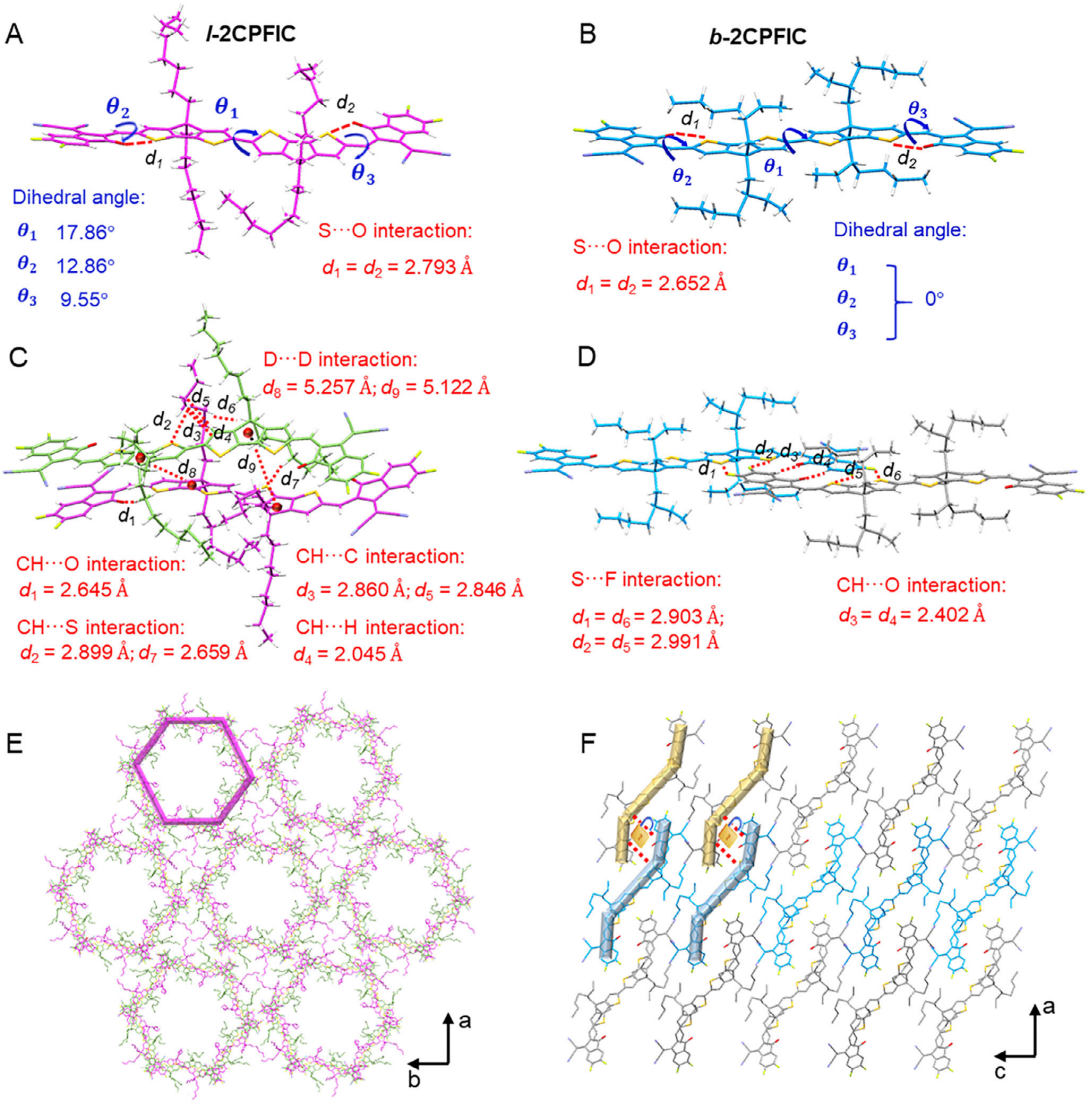
Single‐crystal analysis of *l*‐2CPFIC (recrystallization from CHCl_3_/ CH_2_Cl_2_/hexane) and *b*‐2CPFIC (recrystallization from CHCl_3_/hexane). Single molecule structure and intermolecular interactions of (A, C) two closely contacted *l*‐2CPFIC and (B, D) *b*‐2CPFIC in horizontal directions. (E,F) Molecular stacking in single‐crystals. Colors represent the different conformers.

### 2.4 Theoretical Calculation

To gain a comprehensive understanding of the photophysical and electronic properties of *l*‐2CPFIC and *b*‐2CPFIC, density functional theory (DFT) and time‐dependent density functional theory (TD‐DFT) based on the crystal structure were performed. As displayed in Figure 5A, the lowest unoccupied molecular orbitals (LUMOs) of the two molecules were mainly distributed along the entire backbones while the highest occupied molecular orbitals (HOMOs) dominantly located on the CP cores. Notably, *b*‐2CPFIC exhibited narrower HOMO–LUMO gap (Δ*E*_L‐H_) of 2.067 eV than *l*‐2CPFIC (2.129 eV), reflecting its superior conjugation. QM/MM simulations further confirmed that *b*‐2CPFIC maintained a more planar geometry in the aggregate state, validating that its red‐shifted absorption stemmed from packing‐driven planarization (Figure S38). Furthermore, two essential parameters including singlet‐triplet energy gap (Δ*E*_ST_) and spin‐orbit coupling (SOC, ξ) were calculated to evaluate the ISC rate which decided the ROS generation efficiency in the PDT process. It was obvious that *b*‐2CPFIC possessed a reduced Δ*E*_ST_ value (0.06 eV, 0.1 eV) and an enhanced ξ value (0.21 cm^−1^, 0.45 cm^−1^) compared with *l*‐2CPFIC (Figure 5B). Hence, it was reasonable that *b*‐2CPFIC displayed efficient ROS generation. Moreover, to investigate the essence of the difference in photophysical properties caused by packing difference, Hirshfeld surface analysis was further employed to quantify the intermolecular interaction of crystals. The corresponding fingerprints figure of *l*‐2CPFIC and *b*‐2CPFIC were plotted in Figure 5C [46, 47]. Compared with *l*‐2CPFIC, much stronger intermolecular interaction existed in *b*‐2CPFIC. In addition to dominant C···H/H···C and H···H interactions in both two crystals, non‐negligible S···F/F···S interactions were observed in *b*‐2CPFIC, whose magnitude was stronger distinctly than that in *l*‐2CPFIC. Additionally, the contribution of C···C interaction the *b*‐2CPFIC crystal (0%) was lower compared with that in the *l*‐2CPFIC crystal (5.7%). Further Independent Gradient Model (IGM) analysis revealed that localized S···F atomic pairs act as high‐strength anchors to rigidly lock the adjacent *b*‐2CPFIC backbones, which is entirely absent in *l*‐2CPFIC (Figures S39 and S40). These results demonstrated that the introduction of branched side chain strengthened intralayer intermolecular interaction to promote the formation of absolutely coplanar skeleton, concurrently restricting the intramolecular motion and attenuating π‐π stacking interaction.

**FIGURE 5:**
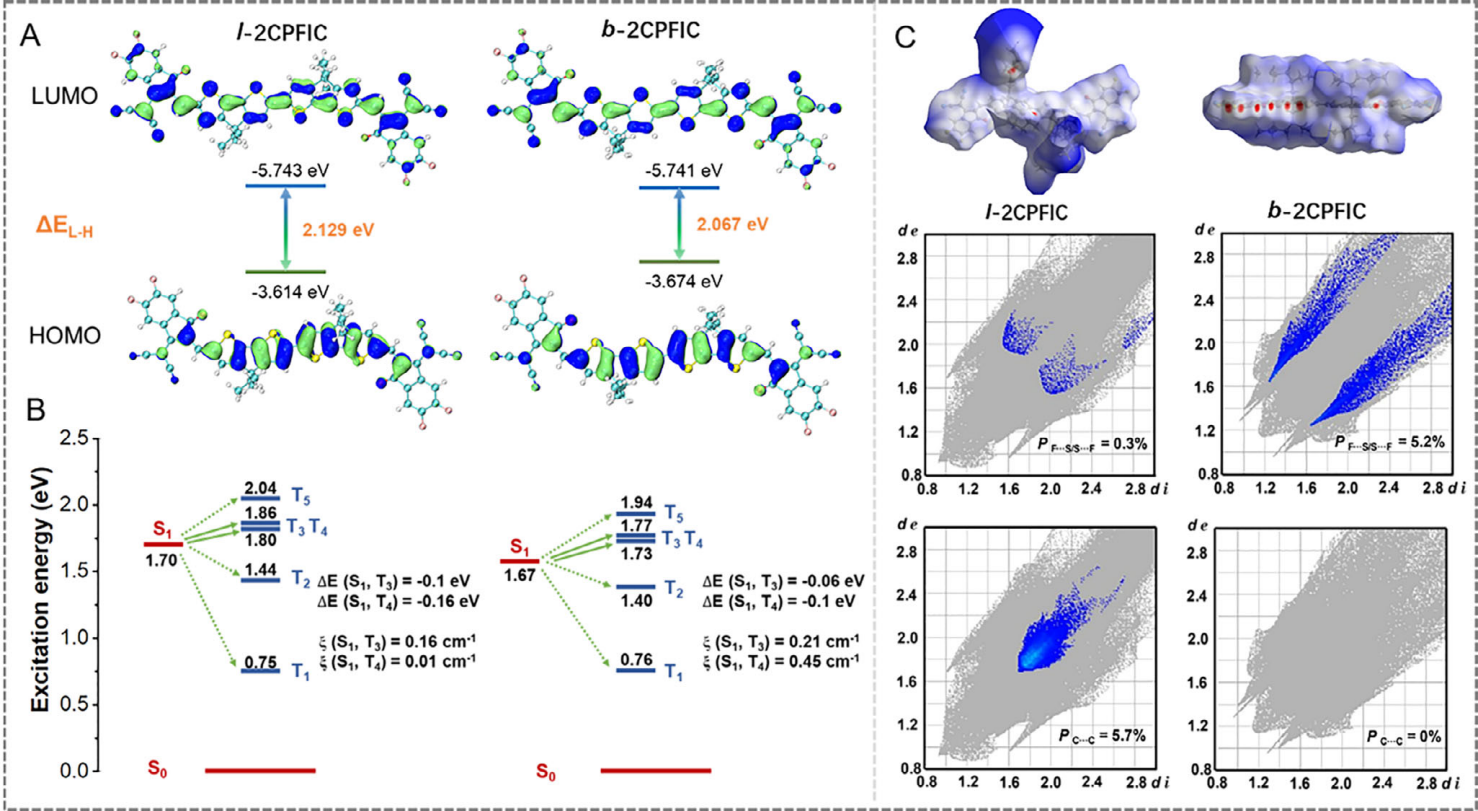
(A) Calculated HOMO‐LUMO distributions of *l*‐2CPFIC and *b*‐2CPFIC based on the crystal structure. *E*_L‐H_ = *E*_LUMO–_ *E*_HOMO_. Isovalue = 0.05. (B) Calculated energy levels, singlet‐triplet energy gaps (∆*E*_ST_) and spin‐orbit coupling constants (ξ) of *l*‐2CPFIC and *b*‐2CPFIC. (C) Hirshfeld surface analysis and fingerprint plot of *l*‐2CPFIC and *b*‐2CPFIC. Blue, weak interactions; white, medium interactions; red, strong interaction.

### 2.5 In Vitro Phototherapy

According to the above analysis of NIR‐II fluorescence, photothermal and ROS generation efficiency, *b*‐3CPFIC NPs with optimal performance were promising for application in the field of multimodal‐ guided phototherapy. The phototheranostic capacity of *b*‐3CPFIC NPs on 4T1 tumor cell was quantitatively evaluated by MTT assay. In the absence of laser irradiation, the cell viability maintained over 80% after 24 h incubation even at a concentration of up to 100 µm. This suggested the good biosafety of the *b*‐3CPFIC NPs. Nevertheless, upon 808 nm laser irradiation for 10 min (0.8 W cm^−2^), dose‐dependent phototoxicity against 4T1 cell was observed and almost 70% of cells were killed at a concentration of 50 µm (Figure 6A). In addition, the cell viabilities of the *b*‐3CPFIC NPs toward NIH‐3T3 normal cell were investigated. As shown in Figure S41, the cytotoxicity toward normal cells were weak regardless of the laser irradiation. The cell viability remained over 90% even at a concentration of 100 µm. These results indicated that the *b*‐3CPFIC NPs not only showed good biocompatibility, but also were efficient in vitro photo therapeutics of tumor cells. To further evaluate the synergistic effect of PDT/PTT, we investigated the PDT and PTT separately, in which 4T1 cell were pretreated with N‐acetylcysteine (NAC), a ROS scavenger, for the individual PTT test or irradiated with 808 nm laser on ice to suppress the temperature raise for PDT treatment. As depicted in Figure 6B, individual PDT and PTT treatments led to approximately 40% and 60% cell mortality, respectively, whereas their combination achieved a superior therapeutic efficacy approaching 90%. To quantitatively evaluate this joint effect under this single‐concentration condition, the combination index (CI) was calculated based on the classic Bliss independence model. The resulting CI value was determined to be 0.86, indicating a positive synergistic trend between PDT and PTT. This potent biological synergism stems from a reciprocal reinforcement within the tumor microenvironment, where PTT‐induced hyperthermia alleviates hypoxia to fuel the PDT, while PDT‐generated ROS compromises heat‐shock proteins to overcome cellular thermal resistance. Additionally, the in vitro ROS generation of NPs was determined using DCFH‐DA. As shown in Figure 6C, 4T1 cells treated with *b*‐3CPFIC NPs and laser irradiation exhibited strong green fluorescence, while no obvious fluorescence was observed in other control groups, implying the effectively ROS generation of NPs in the complex biological system. To further investigate the excellent phototherapy ability of NPs, calcein‐AM (green‐fluorescence live‐cell probe) and propidium iodide (PI, red‐fluorescence dead‐cell probe) co‐staining experiments were conducted. Only the groups treated with *b*‐3CPFIC NPs and laser irradiation revealed obvious red fluorescence while the control, sole light and sole NPs groups showed green fluorescence, indicating the distinguished cytocidal activity and anticancer potential of *b*‐3CPFIC NPs under laser irradiation (Figure 6C).

**FIGURE 6:**
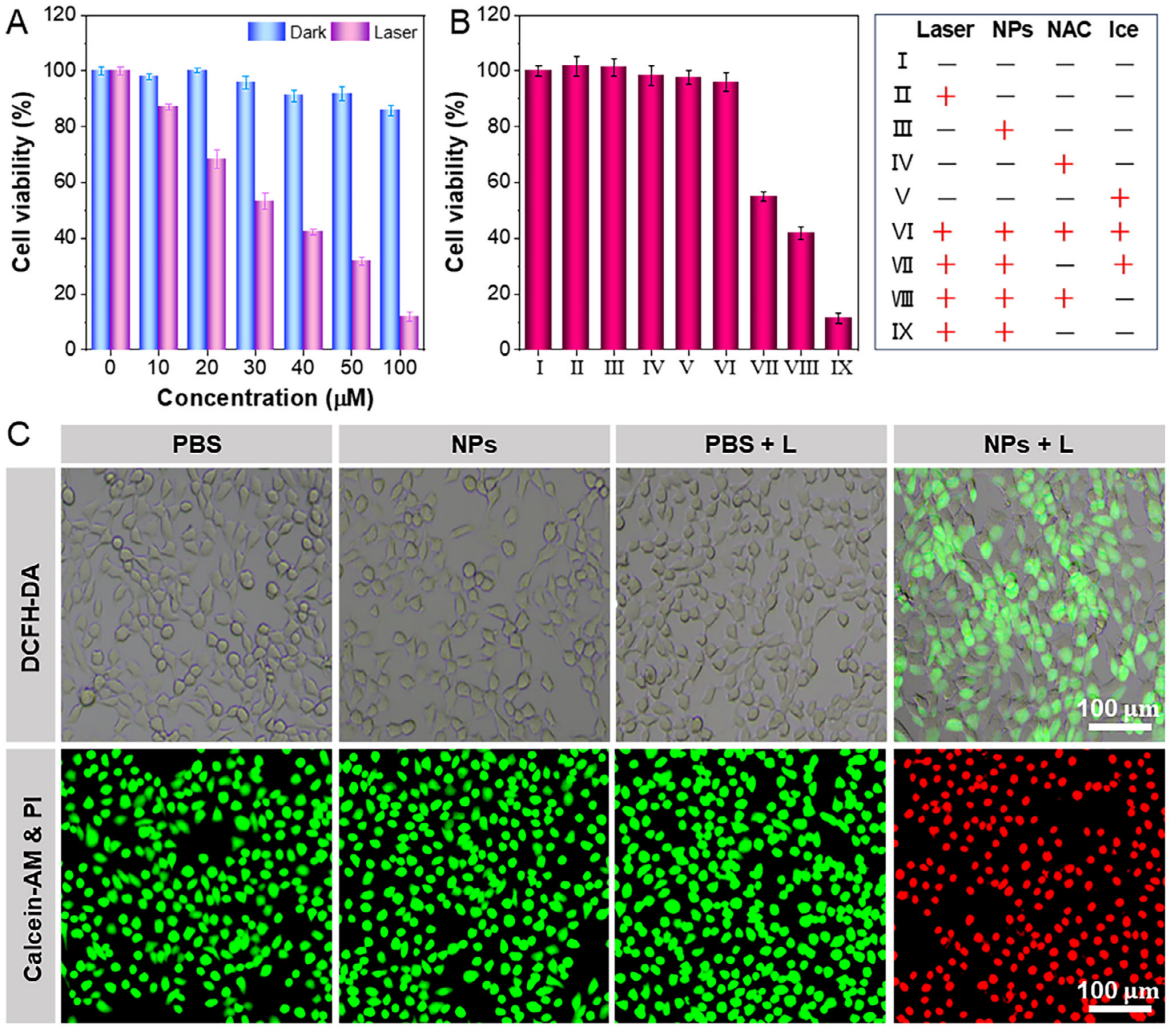
(A) In vitro therapeutic efficacy and biocompatibility of *b*‐3CPFIC NPs (abbreviated as NPs) estimated using MTT. (B) Relative viability of 4T1 cells separated with nine group treated by different experimental conditions (concentration: 100 µm). “+” represented the addition of the corresponding treatment. “‐” indicated the remove of the compound. NAC was utilized to consume the ROS generated by the PDT process and ice was used for cooling to avoid the PTT effect. (C) Intracellular ROS monitoring of 4T1 cells treated with PBS, laser irradiation (L + PBS), *b*‐3CPFIC NPs (NPs), or laser irradiation + *b*‐3CPFIC NPs (NPs + L) using DCFH‐DA probe (808 nm, 0.8 W cm^−2^ for 5 min). Live/dead assays of 4T1 cell after treatment at different conditions (laser: 808 nm, 0.8 W cm^−2^ for 5 min). The green fluorescence from calcein‐AM and red fluorescence from PI represented live cells and dead cells, respectively.

### 2.6 In Vivo Multimodal‐Imaging‐Guided Phototherapy

Motivated by the outstanding fluorescence property and in vitro phototherapeutic efficacy, in vivo multimodal imaging performance of the *b*‐3CPFIC NPs was assessed. First, benefiting from the excellent tissue penetration capability of *b*‐3CPFIC NPs (Figure S42), the in vivo NIR‐II fluorescence imaging (FLI) performance was evaluated using 808 nm laser as excitation. Immediately after tail intravenous injection, the blood vessels in the abdomen and hindlimb were clearly observed from the surrounding background tissue under the LP 1300 (Figure 7A and Figure S43). Detailed position was selected from the abdomen and its diameters were estimated to be 146 µm via FWHM based on the Gaussian fitted curves of the cross‐sectional intensity profiles (Figure 7B).

**FIGURE 7:**
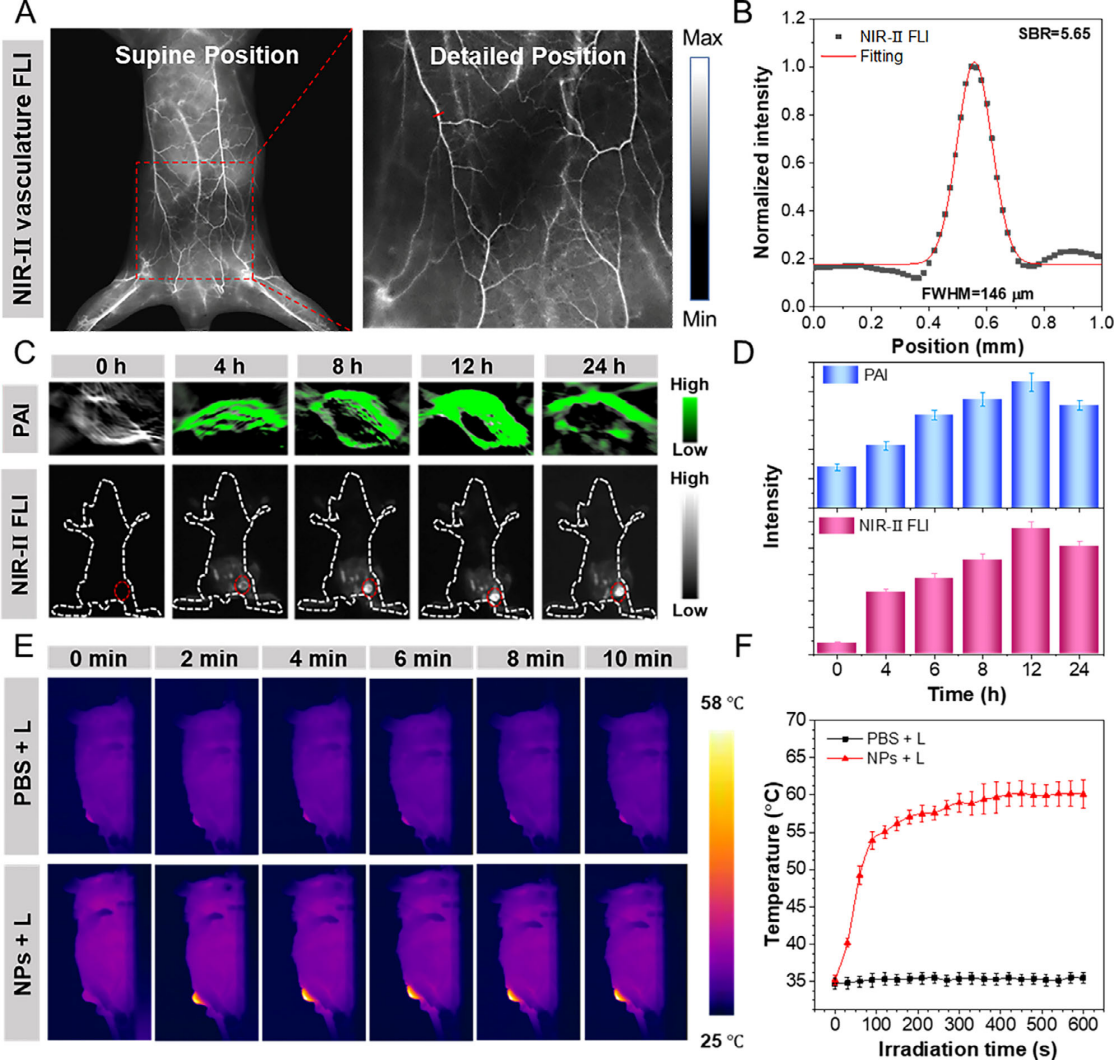
In vivo fluorescence imaging, photoacoustic imaging and infrared thermal imaging. (A) The NIR‐II FLI of blood vessel of mice in supine position 5 min after intravenous injection of *b*‐3CPFIC NPs (100 µL, 100 µm). (B) Cross‐sectional intensity profile along the red line in Figure A with the peak fitted to a Gaussian function (red curve). (C) Images and (D) corresponding signals intensity of PA and NIR‐II FL of tumor‐bearing mice after intravenous injection of *b*‐3CPFIC NPs at different monitoring time. (E) The IR thermal imaging and (F) corresponding temperature variations of tumor sites after injection of PBS, *b*‐3CPFIC NPs under laser irradiation (808 nm, 0.8 W cm^−2^) for different time respectively (mean ± SD, *n* = 3).

The signal‐to‐background ratios (SBR) of the selected area was 5.65, representing a 3.9‐fold enhancement compared to *l*‐3CPFIC NPs (Figure S44), indicating superior in vivo NIR‐II FLI capability. During the short‐term imaging process, spleen was the main organ involved in metabolism (Figure S45). Furthermore, the high‐resolution imaging modality prompted further interest in its tumor imaging. Considering the intense photoacoustic signals of the *b*‐3CPFIC NPs (Figure S46), the accumulation of NPs in the tumor at different time points was detected via photoacoustic imaging (PAI) and NIR‐II FLI. As illustrated in Figure 7C, D, the signal intensities of NIR‐IIa FLI and PAI in the tumor region gradually increased and achieved peak levels after 12 h injection, which verified the good enrichment ability of *b*‐3CPFIC NPs at the tumor site. This highly efficient accumulation is likely driven by a synergy of the EPR effect and an active nanomaterials‐induced endothelial leakiness (NanoEL) mechanism [48, 49, 50]. The signals then slightly decreased after 24 h post‐injection because of metabolism (Figure 7D). The in vivo biodistribution of NPs in the main organs of 4T1 tumor‐bearing mice 24 h‐post injection was determined by NIR‐II fluorescence. It was found that the *b*‐3CPFIC NPs were enriched in liver and kidney, suggesting liver and spleen may participate in their metabolism (Figure S47). The perfect combination of FLI and PAI allowed promoted tissue penetration and better spatiotemporal resolution, which made the *b*‐3CPFIC NPs feasible for precise guidance in next‐generation in vivo tumor therapy. Furthermore, in vivo photothermal performance was evaluated through NIR‐II thermographic imaging of tumor after 12 h post‐injection under laser irradiation. As exhibited in Figure 7E, F, tumor treated with *b*‐3CPFIC NPs demonstrated rapid photothermal conversion, achieving a temperature elevation to 57.0°C within 3 min, significantly surpassing both the concurrent PBS control and the *l*‐3CPFIC NPs‐treated groups under identical irradiation conditions (Figure S48). Collectively, these results had solidly confirmed the excellent NIR‐II FLI/PAI/PTI trimodal imaging ability of *b*‐3CPFIC NPs, which greatly facilitated the subsequent tumor site‐specific synergistic phototherapy with effective decrease of undesired damage to normal tissues.

### 2.7 In Vivo Synergistic Phototherapeutics

Encouraged by the tumor‐enriched multimodal imaging capabilities and exciting in vitro antitumor effect of NPs, their synergistic antitumor efficacy was further evaluated on 4T1‐tumor‐bearing mice in vivo. Tumor‐carrying mice were stochastically divided into four groups (5 mice for each group: I) PBS, II) NPs (100 µm based on *l*‐3CPFIC NPs), III) PBS + L (Laser), and IV) NPs + L (100 µm based on *l*‐3CPFIC NPs plus 808 nm laser irradiation). According to the schematic diagram of the treatment process (Figure 8A), 4T1 tumor cells were inoculated to construct subcutaneous tumors and then grew for 8 d before the execution of treatment. Following injection with NPs (or PBS) through tail vein, the tumor sites of mice were exposed to the 808 nm laser at 0.8 W cm^−2^ for 5 min after 12 h accumulation. During the 16‐day treatment, apart from monitoring the tumor size via Vernier caliper, the real‐time quantitative monitoring of luciferase‐labeled 4T1 cells was performed to determine tumor growth (Figure 8B).

**FIGURE 8:**
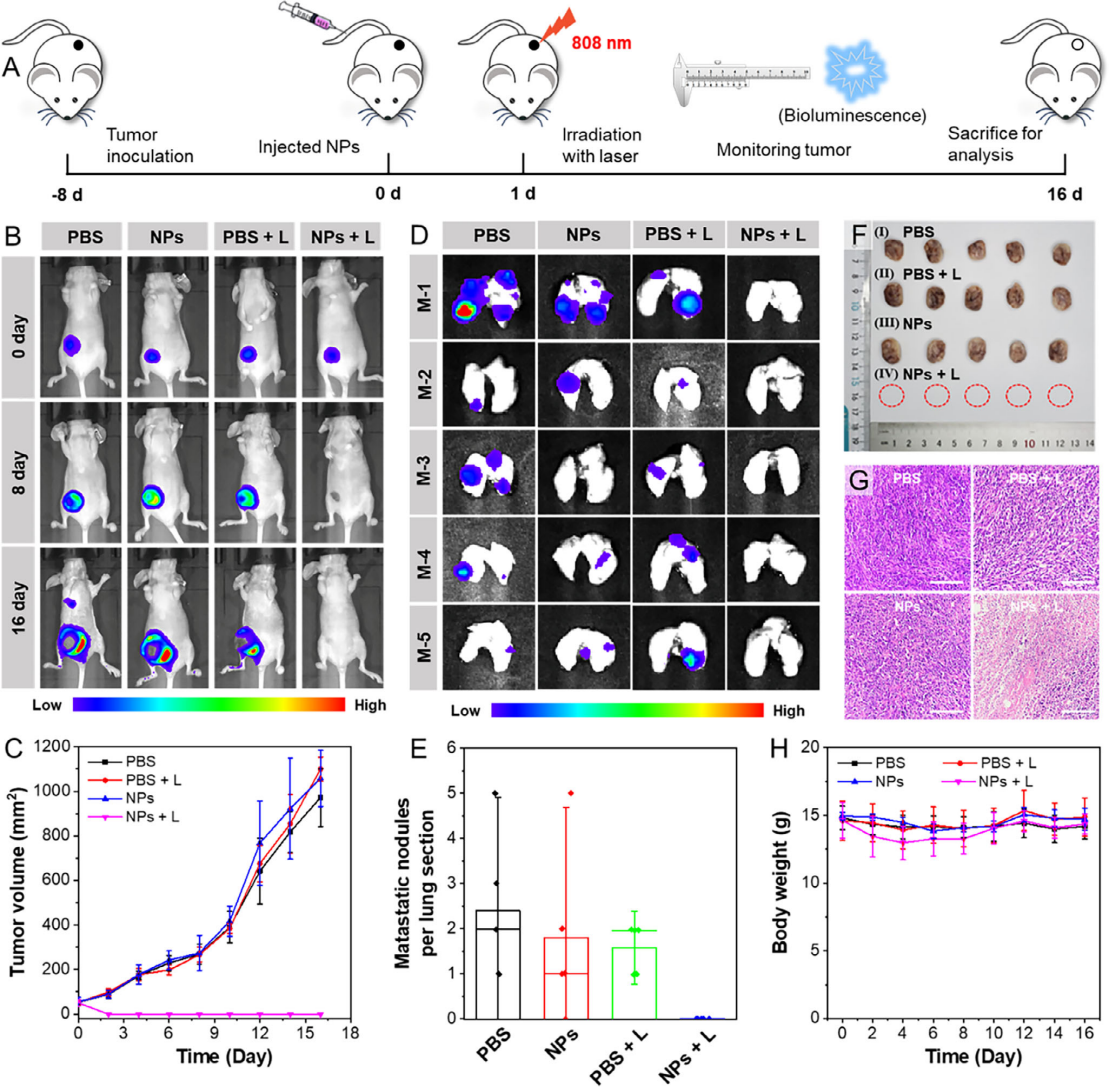
In vivo PDT and PTT combination therapy. (A) Schematical diagram of the experiments process in vivo. (B) Bioluminescent images of 4T1 tumor‐bearing mice after different treatments for 0, 8, and 16 d (*n* = 3). Left to right: PBS, *b*‐3CPFIC NPs, PBS + laser, *b*‐3CPFIC NPs + laser. Laser conditions: 808 nm, 0.8 W cm^−2^. (C) Tumor growth profile of tumor‐bearing mice in different treatment groups. (D) Bioluminescent images and (E) statistical numbers of lung metastatic nodules in tumor‐bearing mice after different treatments. (F) Tumor photographs from different treatment groups. (G) H&E staining analysis of tumors tissues in different groups. (H) Body weight curves of tumor‐bearing mice in different treatment groups.

Compared with the control group, the tumors in mice treated with NPs plus laser irradiation were completely eliminated at as early as day 2 after treatment without any regrowth or recurrence even completely ablated in the 16 d post‐treatment. This revealed the excellent synergistic PDT/PTT phototherapeutic efficacy of NPs. In sharp contrast, the tumor in other three control groups grew rapidly during the whole observation period, and the tumor volumes had no obvious difference as compared with each other, suggesting that laser irradiation and NPs treatments did not affect the tumor growth when administered alone (Figure 8B, C). Moreover, the remarkable reduction of lung metastatic nodules in “NPs + L” group and the photographs of ex vivo tumors demonstrated the excellent capability of NPs for tumor‐growth inhibition both primary tumor elimination and distant metastasis prevention (Figure 8D–F). This prominent anti‐metastatic efficacy can be rationalized by the effective destruction of the primary lesion to block tumor cell intravasation, together with the well‐established systemic antitumor immunity triggered by local phototherapy [51, 52, 53]. To further confirm the antitumor effects, the excised tumor slices were examined by hematoxylin and eosin (H&E) staining analysis. Obvious prominent necrosis and cell apoptosis were observed in the group of “NPs + L”, while no remarkable lesion was found in other control group (Figure 8G). These results indicated that *b*‐CPFIC NPs could serve as a synergistic PDT/PTT agent with excellent therapeutic effect, requiring only a single injection and one‐time laser irradiation.

Subsequently, the toxicity of *b*‐CPFIC NPs was also estimated carefully. During the whole process, the body weight of mice in all groups showed inconspicuous changes (Figure 8H), which validated the good biocompatibility of the NPs. In addition, the blood routine examination results exhibited that there were no statistical differences between the treatment group and other control groups (Figure S40), which demonstrated that the NPs induced negligible infection and inflammation in the mice. Beyond that, H&E staining analysis of major organs collected from the mice after the 16 d treatments. No significant damage to the main organs, including the heart, liver, spleen, lung, and kidney, was observed (Figure S49). All of these results confirmed that the NPs could be applied as a safe and powerful theranostic agents for synergistic PDT/PTT anticancer in vivo.

## 3 Conclusion

In summary, a bio‐inspired side‐chain branching strategy derived from the protein β‐sheet motif was developed to orchestrate a synergistic intra‐/intermolecular NoCL network, successfully achieving perfectly planar A–D–A type NIR‐II phototheranostic aggregates. Crystallographic analysis and theoretical calculations demonstrated that the stereoregular branched topology effectively mediates the molecular packing modes to bypass the twisted aggregate conformations typical of the linear counterparts. By strategically regulating the aggregate‐state steric environment, this branched architecture successfully preserves the intrinsic intramolecular NoCL while activating robust intermolecular S···F interactions. This precise steric‐electronic cooperative effect within the interaction‐active framework locks the backbone into an absolute planar geometry, leading to a 3‐fold fluorescence brightness improvement, a 5‐fold ROS generation efficiency boost, and a 1.38‐fold photothermal enhancement. These photophysical optimizations and their underlying mechanisms were unambiguously decoded by fs‐TAS and validated across two distinct framework skeletons (2CPFIC and 3CPFIC). Capitalizing on these exceptional properties, the optimized *b*‐3CPFIC NPs were successfully applied for NIR‐II and PA imaging‐guided synergistic PDT/PTT. Consequently, this work establishes a conceptually generalizable side‐chain‐mediated dual‐NoCL strategy for high‐performance organic phototheranostics aggregates, governed by three key design principles: an interaction‐active backbone with heteroatom docking sites, branched chains for aggregate‐state steric control, and a delicate steric‐electronic balance for absolute planarity locking. This structure–conformation–performance paradigm provides deep insights into aggregate science and offers a powerful methodology for breaking conventional photophysical trade‐offs.

## Author Contributions

**Cheng Lu**: conceptualization, methodology, investigation, Writing – original draft, formal analysis, and data curation. **Bo Wu**: validation and software. **Qian Jia**: methodology and data curation. **Jacky W. Y. Lam**: writing – review and editing, project administration, validation. **Zheng Zhao**: validation, visualization. **Ben Zhong Tang**: funding acquisition, project administration, supervision, writing – review and editing, and conceptualization. **Chao Li**: investigation and methodology. **Zhongliang Wang**: visualization, validation, and methodology. **Teng‐Teng Chen**: funding acquisition, visualization, and validation. **Ryan T. K. Kwok**: project administration. **Tie‐Gen Chen**: funding acquisition and validation. **Jianwei Sun**: project administration.

## Conflicts of Interest

The authors declare no conflicts of interest.

## Supporting information




**Supporting File**: advs76688‐sup‐0001‐SuppMat.docx.

## Data Availability

The data that support the findings of this study are available from the corresponding author upon reasonable request.
