# Supplementary material for: Engineering Synergistic Intra‐/Intermolecular Conformational Locking via Side‐Chain Branching: A β‐Sheet‐Inspired Strategy for Planar NIR‐II Phototheranostic Aggregates
Source: Adv Sci (Weinh). 2026 Jul 24:e76688. Online ahead of print. doi: 10.1002/advs.76688 (PMC13397326; doi:10.1002/advs.76688)
Supplement: Supplementary file 1 — Supporting File: advs76688‐sup‐0001‐SuppMat.docx. [file ADVS-9999-e76688-s001.docx]

Supporting Information

**Engineering Synergistic Intra-/Intermolecular Conformational Locking via Side-Chain Branching: A β-Sheet-Inspired Strategy for Planar NIR-II Phototheranostic Aggregates**

*Cheng Lu,^[a],#^ Qian Jia,^[b],#^ Bo Wu,^[c],#^ Chao Li,^[d]^ Ryan T. K. Kwok,^[a]^ Tie-Gen Chen,^[e] ,^* Jianwei Sun,^[d]^ Zheng Zhao,^[f]^ Teng-Teng Chen,^[d],^* Zhongliang Wang,^[b],^* Jacky W. Y. Lam,^[c],^* and Ben Zhong Tang^[a][f],^**

[a] Dr. C. Lu, Prof. R. Kwok, Prof. J. Lam, and Prof. B. Z. Tang

Department of Chemistry, Hong Kong Branch of Chinese National Engineering Research Center for Tissue Restoration and Reconstruction, Division of Life Science and State Key Laboratory of Molecular Neuroscience, The Hong Kong University of Science and Technology, Kowloon 999077, Hong Kong, China
* Correspondence E-mail: chjacky@ust.hk (Prof. J. W.Y. Lam) and tangbenz@ust.hk (Prof. B. Z. Tang).

[b] Prof. Z. Wang and Prof. Q. Jia

Lab of Molecular Imaging and Translational Medicine (MITM), Engineering Research Center of Molecular and Neuro Imaging, Ministry of Education, School of Life Science and Technology, Xidian University & International Joint Research Center for Advanced Medical Imaging and Intelligent Diagnosis and Treatment, Xi’an, Shaanxi, 710126, China
* Correspondence E-mail: wangzl@xidian.edu.cn (Prof. Z. Wang)

[c] Prof. B. Wu

School of Environmental and Chemical Engineering, Wuyi University, Jiangmen, Guangdong, 529020, China

[d] Prof. T.-T. Chen, Prof. J. Sun and Dr. C. Li

Department of Chemistry, The Hong Kong University of Science and Technology, Kowloon, Hong Kong 999077, China
* Correspondence E-mail: tengtengchen@ust.hk (Prof. T. Chen)

[e] Prof. T.-G. Chen

Zhongshan Institute for Drug Discovery, Shanghai Institute of Materia Medica, Chinese Academy of Sciences, Zhongshan, 528400, China; * Correspondence E-mail: chentiegen@simm.ac.cn

[f] Prof. Z. Zhao, and Prof. B. Z. Tang

Guangdong Basic Research Center of Excellence for Aggregate Science, School of Science and Engineering, Shenzhen Institute of Aggregate Science and Technology, The Chinese University of Hong Kong, Shenzhen (CUHK-Shenzhen), Guangdong 518172, China. E-mail: tangbenz@cuhk.edu.cn (Prof. B. Z. Tang)

# These authors contributed equally to this work.

Experimental Section/Methods

**Materials.** All the chemicals and reagents were purchased from Energy-Chemical, SigmaAldrich, J*&*K without additional treatment before use. Pluronic F127 was purchased from Xi’an ruixi Biological Technology Co., Ltd. The biological chemical reagents containing the ROS indicators 9,10-anthracenediyl-bis(methylene)-dimalonic acid (ABDA), 2', 7'-Dichlorodihydrofluorescein diacetate (DCFH-DA), Dihydrorhodamine 123 (DHR123) and hydroxyphenyl fluorescein (HPF) were obtained from Aladdin Co., Ltd. Calcein acetoxymethyl ester (Calcein-AM), propidium iodide (PI), and Thiazolyl blue tetrazolium bromide (MTT) was purchased from Beyotime biotechnology Co., Ltd (Shanghai, China). Deionized (DI) water (18.2 MΩ·cm at 25 C) was produced by a Milli-Q water purification system (Millipore).

**Measurements.** NMR spectra were measured using a Bruker 400 MHz NMR spectrometer with CDCl_3_. The high-resolution mass spectra (HRMS) were collected with GCT Premier CAB 048 mass spectrometer in MALDI-TOF mode. UV absorption spectra were recorded using a SHIMADZU UV-2600i Spectrophotometer. Fluorescence spectra were obtained using Edinburgh Instruments FLS1000. The size distribution and average size of these nanoparticles (NPs) were performed by DLS using a Zetaplus potential analyzer. Confocal laser scanning microscope (CLSM) images were obtained using an Leica confocal laser scanning microscope. TEM images were captured by using a FEI Tecnai F20 microscope (accelerating voltage of 200 kV). All the NIR-II images were captured by using a NIR-II live animal imaging system (Series II 900/1700-H, China). DFT calculations were carried out by using the B3LYP/6G(d), Gaussian 09 package. Temperature change was monitored by an FLIR A300-Series thermal imaging camera (FLIR System). Photodynamic and photothermal experiments were conducted using an 808 nm infrared semiconductor laser (CNI laser, MDL-H-808-5W). The output power of the lasers was measured with a LP100 laser power meter (Changchun New Industries Optoelectronics Technology Co., Ltd., China). The in vivo NIR-II fluorescence was imaged by full-spectrum in vivo fluorescence imaging system (Suzhou NIR-Optics Technology Co., Ltd) under 808 nm laser excition (30 mW cm^-2^, 100 ms). PA studied by MSOT InVision 128 imaging system (iThera Medical). The blood biochemistry parameters were collected by the fully automatic biochemical analysis (Chemray 240).

**Preparation of NPs.** *l*-2CPFIC (2 mg) or *b*-2CPFIC (2 mg) or *l*-3CPFIC (2 mg) or *b*-3CPFIC (2 mg) in 1 mL tetrahydrofuran (THF) was added in a solution of Pluronic F127 (12 mg) in DD water (9 mL). The mixed solution was dealed by an Ultrasonic Cell Disintegrator for 10 min, and then the solution was stirred overnight to volatilize THF from the solution. The NPs solution was filtered with a 0.22 *μ*m membrane to remove the insoluble particles and concentrated by ultrafiltration centrifuge tube.

**Theoretical Calculation of Fractional Free Volume (FFV)** To quantitatively substantiate the molecular packing density in the aggregated state, the FFV of both l-2CPFIC and b-2CPFIC aggregates was theoretically calculated using the Multiwfn program. The calculation was based on a periodic boundary condition unit cell model, with the crystallographic information file (CIF). Following the promolecular density-based grid occupancy method, the unit cell was partitioned into discrete grid points. A grid point was considered occupied if its promolecular density exceeded a preset threshold (threshold for defining free space was set to 0.0001 a.u., corresponding to approximately 0.001 e^-^/bohr^3^); otherwise, it was regarded as free space. The free volume was then obtained by multiplying the number of free grid points by the volume of a single grid point. The FFV was defined as the free volume divided by the total cell volume (FFV = free volume / total cell volume). For the analysis, the periodic system cavity analysis module was utilized. Following the recommended practice for periodic systems, a grid spacing of 0.2 Å was employed to balance computational efficiency and volumetric accuracy, with the promolecular density threshold set as described. The software outputs the total cell volume (Å³), the free volume (Å³), and the resulting FFV (as a percentage of the total space).

**Determination of fluorescence quantum yield (QY).** The fluorescence quantum yields of *l*-2CPFIC NPs, *b*-2CPFIC NPs, *l*-3CPFIC NPs and *b*-3CPFIC NPs were determined from the relative QY by comparing with the IR-26 (*Φ* = 0.5% in 1,2-dichloroehane (DCE)), which is a reliable and most commonly used NIR-II QY reference. ^1-6^ The quantum yields were calculated based on the equation:

$$\Phi_{sample}=\Phi_{ref}\times\frac{{Slope}_{sample}}{{Slope}_{ref}}\times\left( \frac{n_{sample}}{n_{ref}} \right)^{2}$$

where subscripts *sample* and *ref* represent sample and reference, respectively. *Slope* is the slope of the integrated fluorescence intensity against the absorbance plot (linear fitting). *n* is the refractive index of the solvent (*n*_water_ = 1.33*, n_DCE_* = 1.44*)*. The maximal absorptions of all samples were carefully controlled to lower than 0.1 for all measurements to eliminate self-quenching or re-absorption/re-emission issues. The sample and reference were excited by the same laser (808 nm). The integrated fluorescence intensity of sample and reference was measured in 850-1400 nm.

**Photothermal properties of NPs.** The photothermal conversion efficiencies of *l*-2CPFIC NPs, *b*-2CPFIC NPs, *l*-3CPFIC NPs and *b*-3CPFIC NPs were calculated according to the reported method.^7-11^ Taking the *b*-3CPFIC NPs as the example, with a concentration of 10 μM was irradiated with an 808 nm laser light (0.5 W cm^-2^) for 5 min. The laser light was turned off, and the NPs solution cooled naturally for 12 min. During this period, an infrared thermal imager was used to measure the in-situ temperature.

The photothermal conversation efficacy (*η*) was calculated by equation:

$\eta=\frac{hS\left( T_{max}-T_{surr} \right)-Q_{0}}{I\left( 1-{10}^{{-A}_{808}} \right)}$ (1)

*h* represents the heat transfer coefficient; *S* represents the surface area of the Eppendorf Micro Test Tubes. *T*_Max_ and *T*_Surr_ denote the steady-state maximum temperature and the ambient temperature of the surroundings, respectively. *t* represents the time it takes for *T_max_* to cool to room temperature, *Q_0_* represents the energy input by the same solvent without NPs in the same quartz cuvette after the same laser irradiation, and it is typically 14 mW for pure water. *I* is the incident laser power (0.5 W cm^-2^). 𝐴_808_ is the absorbance of the sample at 808 nm. The value of *hs* can be calculated by the follow equation:

$hs=\frac{\sum{m_{i}C}_{pi}}{\tau_{s}}$ (2)

$\tau_{s}=\frac{t}{-In\theta}$ (3)

$\theta=\frac{T-T_{surr}}{T_{max}-T_{surr}}$ (4)

$Q_{0}=hs\left( T_{max}-T_{surr} \right)$ (5)

where *τ*_s_ is the time constant for system heat transfer, *C* approximate to the specific heat capacity of water, *m* represented the mass of the solution (g). $\sum{m_{i}C}_{pi}$are heat capacity of deionized water used to dissolve the *b*-3CPFIC NPs. *τ*_s_ can be calculated according to formula (3) and (4). *θ* is the ratio of *T*-*T*_surr_ to *T_max_*-*T*_surr_ (4). According to the recorded temperature values at different times, the change in temperature is calculated, and the linear relationship between time and -ln *θ* is drawn, and the value of *τ*_s_ can be calculated according to the slope. The PCE of other NPs can be calculated according to the same method as above.

**Total reactive oxygen species (ROS) detection by dichlorodihydrofluorescein (DCFH).**^12-14^ DCFH-DA is used as the probe to measure the total ROS generation of these NPs under 808 nm laser radiation. Activation of DCFH-DA involved dissolving 2.44 mg of the compound in 5 mL ethanol solution, followed by mixing 0.5 mL of the DCFH-DA ethanol solution (10 mM) with 2 mL of 100 mM sodium hydroxide solution for 30 min activation period. Then, 10 mL of PBS solution was introduced to abtain the DCFH solution. In the experiments, PBS buffer solution containing NPs (10 μM) in quartz cuvettes were mixed with DCFH (final concentration 40 μM). The mixed solution was irradiation by laser at 0.5 W cm^-2^ for a period of 60 s. The fluorescence signal of indicator was monitored in a range of 500-600 nm with the excitation wavelength at 488 nm. The fluorescence intensity at 525 nm was recorded to indicate the general ROS generation rate.

**Crystal growth.** *b*-2CPFIC (0.5 mg) was dissolved with Chloroform (0.5 mL) in a 5 mL vial, then 0.2 mL Dichloromethane and 0.5 mL Hexane were added into the vial, shaking thoroughly to mix them well and sealed with sealing membrane. The container was left undisturbed at room temperature until the growth of the crystals could be observed (10 ~ 15 days). In addition, *l*-2CPFIC crystals were grown in a similar way to *b*-2CPFIC, except for growth temperature. The crystals were obtained in the refrigerator (4 $℃)$ with slower diffusion rate. Crystallographic and structural refining data for *l*-2CPFIC and *b*-2CPFIC are presented in **Table S2**.

**Molecular dynamics (MD) simulations.** For *b*-2CPFIC and *l*-2CPFIC, 18 molecules were first placed in a cubic box with a length of 10 nm. The box was then subjected to energy minimization using a steepest descent algorithm. A 20 ns NPT ensemble simulation (P = 1 atm and T = 298.15 K) was performed to obtain the conformation of nanoparticle. The system then was solvated in a water box (about 30000 water molecules). Energy minimization was first conducted to obtain energetically favorable conformations. Subsequently, an NPT ensemble equilibration simulation was performed under ambient conditions (P = 1 atm, T = 298.15 K) to stabilize the system. Following equilibration, a 20-nanosecond production molecular dynamics simulation was carried out for trajectory sampling and subsequent statistical analysis.^15^

Simulation Parameters:

Pressure control: Parrinello-Rahman barostat.

Temperature control: V-rescale thermostat.

Periodic boundary conditions (PBC) applied in all directions.

Force fields: GAFF for molecules in nanoparticles, and OPLS-AA with SPCE water model for the aqueous environment.

Electrostatics: PME for long-range, 12 Å cutoff for short-range.

Constraints: LINCS for hydrogen bonds, time step = 1 fs.

**Theory calculations.** Free single molecule calculations in solution: Ground-state (S₀) and excited-state (S₁ and T₁) geometry optimizations were performed using the Gaussian 16 package^16^ at the B3LYP-D3/6-31G(d) level. The polarizable continuum model (PCM) was employed to simulate the tetrahydrofuran (THF) solution environment. Based on the optimized stable geometries, vertical excitation energies were calculated using the same DFT functional (B3LYP-D3) with the basis set upgraded to 6-31G(d,p).

Absorption calculations of crystals: the absorption energy level of crystals is calculated with ORCA 5.0 program based on the measured single crystals structure. In order to speed up the calculation, the QM-QM mode was used. The conjugated fragment and the nearby carbon chain were calculated using the b3lyp-D3/def2-TZVP method, while the distant carbon chain part was calculated using the fast XTB method.

Emission calculations of crystals: Optimization of excited-state conformations of crystals were performed with the combined quantum mechanics and molecular mechanics (QM/MM) method using the ONIOM model of the Gaussian 16 package. Crystal systems were constructed based on clusters of molecules cut from single crystals. The central molecule was used as a QM layer for photophysical property calculations at B3LYP-D3/6-31G(d) level and the surrounding molecules acted as the environment and were treated using a universal force field (UFF). Due to resource and technology limitations, the optimized T1 stable conformation was used to represent the optimized S1 stable conformation, because the stable conformation of T1 can be calculated by optimizing the ground state of the triplet system which can usually achieve convergence. Based on the optimized excited state stable conformation, the photophysical properties of the excited state of the crystal were further calculated at the B3LYP-D3/def2-TZVP level with QM/MM method of ORCA 5.0 program, including vertical excitation calculations and SOC matrix elements.

All isosurface maps of the frontier molecule orbitals were analyzed by Multiwfn 3.8(dev) program and rendered by VMD 1.9.3 program.^17-19^ The Hirshfeld surfaces and decomposed fingerprint plots were mapped using Crystal Explorer 17.5 package.^20^ The independent gradient model based on Hirshfeld partition (IGMH) method in Multiwfn 3.8(dev) program was employed to study the weak intermolecular interactions.

**In intra cellular ROS detection**. To detect the total ROS generation in cell, 4T1 cell were incubated with 10 μM of *b*-3CPFIC NPs for 6 h followed by incubation with 10 μM DCFH-DA for 30 min. After being washed by PBS buffer for three times, cells were irradiation with 808 nm laser at power density of 0.5 W cm^-2^ for 10 min. Then, the fluorescence was immediately observed using CLSM with the excitation wavelength of 488 nm, and emission collection wavelength was 510 ~ 550 nm.

**Cell viability was evaluated by MTT assay.** To evaluate the cytotoxicity of *b*-3CPFIC NPs, 4T1 cell with a concentration of 5×10^6^ cells in 100 μL of medium (supplemented with 10% FBS and 1% Penicillin-Streptomycin) per well were carefully seeded in fresh 96-well plates and were incubated for 24 h at 37$℃$ in 5% CO_2_. Then the fresh medium with NPs concentration equivalent to 0, 10, 20, 30, 50, and 100 μM was injected into suitable well to replace the original medium and contiune incubated 20 h. After the cell uptake the NPs adequately, the cells were applied 808 nm laser (0.8 W cm^-2^) irradiation for 10 min (or not treated corresponding to the dark condition). Then the MTT assay was used to test the cell viability. Cell viability was determined by using the given formula:

$Cell viability (\%)=\frac{Absorbance of treated cells}{Absorbance of untreated cells}\times100\%$

In order to investigated the PDT efficency, the ice-bath was ultilized during the laser irradiation period to prevent the excessive temperature. Furthermore, Vitamin C (Vc, μM) was added to the medium and incubating the cell at 37$℃$ for 2 h before replacing with NPs medium to study the only PDT activity. All the results were presented as average ± SD (n = 5).

**Evaluation of Synergistic PDT/PTT Effect based on the classic Bliss independence model.** To quantitatively evaluate the cooperative and synergistic therapeutic outcomes of the combined photodynamic therapy (PDT) and photothermal therapy (PTT) at the designated concentration, the combination index (CI) was determined based on the classic Bliss independence model.^21-22^ The CI value based on cell inhibition rates (E) was calculated according to the following equation:

$$CI={E_{PDT}+E_{PTT}-E_{PDT}\times E_{PTT}}/(S_{PDT+PTT})$$

Where *E*_PDT_, *E*_PTT_, and *E*_PDT+PTT_ represent the experimental cell inhibition rates of individual PDT, individual PTT, and the combined PDT/PTT treatment, respectively. The criteria for evaluating the combined therapeutic relationship are categorized as follows:

CI < 1.0 indicates a synergistic effect.

CI = 1.0 indicates an additive effect

CI > 1.0 indicates an antagonistic effect.

**Live-dead cell staining.** 4T1 cell with a density of 5×10^5^ cell mL^-1^ were seeded in confocal dishes (35 mm) and pre-incubated for 24 h. Then the prepared NPs medium was added to replace the original medium and incubated for 12 h. The cells were subjected to dark or laser irradiation (808 nm, 0.8 W cm^-2^, 10 min) and were further cultured for 4 h. Finally, the cell was staining with Calcein-AM and PI mixture for 30 min and was washed with PBS twice for the next fluorescence imaging. The corresponding fluorescence signal was acquired via inverted fluorescent microscope. (Calcein-AM: *λ*_ex_: 460 – 495 nm, *λ*_em_: > 510 nm; PI: *λ*_ex_: 510 – 550 nm, *λ*_em_: > 575 nm).

**In vivo NIR-II fluorescence imaging of blood vessels.** Animals received care in accordance with the Institutional Animal Care and Use Committee of the Fourth Military Medical University (approval number: 20220290). Upon intravenous injection of *b*-3CPFIC NPs (200 μL, 0.5 mM), the fluorescence imaging of the representative mouse was immediately collected by a small animal imaging system with different long-pass filters (900, 1000, 1100 and 1300 nm).

**Tumor model and imaging.** Animals received care in accordance with the Institutional Animal Care and Use Committee of the Fourth Military Medical University (approval number: 20220290). After being acclimated and tested for infectious diseases for 1 week, 4-week-old BALB/c mice were subcutaneously injected with 4T1 cells (1×10^7^ cells each mouse) at the flank region. After about one week, mice with tumor volumes of about 50-100 mm^3^ were randomized into treatment groups. The tumor size was calculated using the following formula: Volume = (Length×Width^2^)/2. For time-dependent NIR-II fluorescence imaging of 4T1-tumor-bearing mice, the mice were anesthetized with isoflurane. Next, the PBS solution containing *b*-3CPFIC NPs (200 μL, 0.1 mM) was intravenously injected into the mouse. After injection, the mouse was imaged with the small anima imaging system at designated time points with 1300 nm long-pass filters. Meanwhile, the photoacoustic imaging signal at 0, 2, 8, 12, and 24 h post injection was collected via real-time multispectral optoacoustic tomographic (MSOT) imaging system (in Vision 128, iThera Medical GmbH, Neuherberg, Germany). This system incorporates a cylindrically focused curved-array transducer comprising 128 elements arranged in 270° arc configuration with uniform angular distribution. The transducer operates within a frequency bandwidth of 7.5 MHz (peak sensitivity at 5 MHz) and is orthogonally aligned relative to the z-axis, enabling high-resolution cross-sectional imaging. The integrated motorized translation stage facilitates z-axis scanning for comprehensive whole-body imaging. Optical illumination is achieved through a bifurcated fiber bundle that splits the laser output into ten slit-shaped light guides (five per lateral aspect of the transducer), providing 360° full-ring illumination of the imaging plane.

In *in vivo* photoacoustic imaging experiments, PA signal acquisition is performed with the following parameters: Excitation wavelength: 690-900 nm; Repetitive frequency of light pulse: 10Hz; Pulse width of light: 10 ns; Pulse light energy: 30 mJ; Sampling frequency: 40 MHz.

**In vivo phototherapy.** When the tumor reached 50-100 mm^3^, 4T1-luc tumor-bearing mice were randomly divided into four groups (n = 5): I) Control group, the mice were only intravenously injected with 200 μL PBS; III) Laser group, the mice were intravenously injected with 200 μL PBS and then irradiated with 808 nm laser (0.8 W cm^-2^, 30 min) 24 h after the injection; III) NPs group, the mice were only intravenous injected with 200 μL *b*-3CPFIC NPs; IV) NPs + Laser group, the mice were intravenously injected with 200 μL *b*-3CPFIC NPs and then irradiation with 808 nm laser (0.8 W cm^-2^, 30 min) 24 h after the injection. After the tail vein injection of NPs, the tumor size and body weight in each mouse were measured every 2 days for a period of 16 days. The relative tumor volumes were calculated for each mouse as *V*/*V*_0_ (*V*_0_ was the tumor volume when the treatment was initiated). In addition, to study the photothermal conversion of *b*-3CPFIC NPs in *vivo*, the temperature changes of the tumor site of mice in group Ⅰ$-$Ⅳ upon exposure to the laser was monitored with the near-infrared thermal imager. After the 16-day treatment, all the mice were sacrificed. Their main organs (heart, liver, spleen, lung, and kidney) were dissected for tissue slicing and staining, and their tumors were excised for the visualized picture.

**Histological analysis.** The tumor tissues were isolated from mice and then were fixed in 4% neutral buffered formalin. According to the routine procedure, fixed tumor tissues were processed with paraffin and sectioned. Then stained with H&E for the final microscope observation.

**Blood routine test.** The blood in tumor-bearing mice after 16-day different treatment was collected and the blood routine test was conducted on the automatic hematology analyzer (DF52Vet, Dymind Biotech., China).^23^

Synthesis and characterization

**The synthesis procedure of *l*-2CPFIC, *b*-2CPFIC, *l*-3CPFIC and *b*-3CPFIC.** *l*-2CPFIC and *l*-3CPFIC were synthesized according to the method reported by Liʼs work. The synthesis procedure of *b*-2CPFIC and *b*-3CPFIC was exhibited in the Scheme S1. Herein, the synthesis detail of *b*-2CPFIC and *b*-3CPFIC is same as the synthesis procedure of *l*-2CPFIC and *l*-3CPFIC, respectively. Therefore, we do not elaborate the synthesis step and only exhibit the NMR spectra of related compounds.^24^


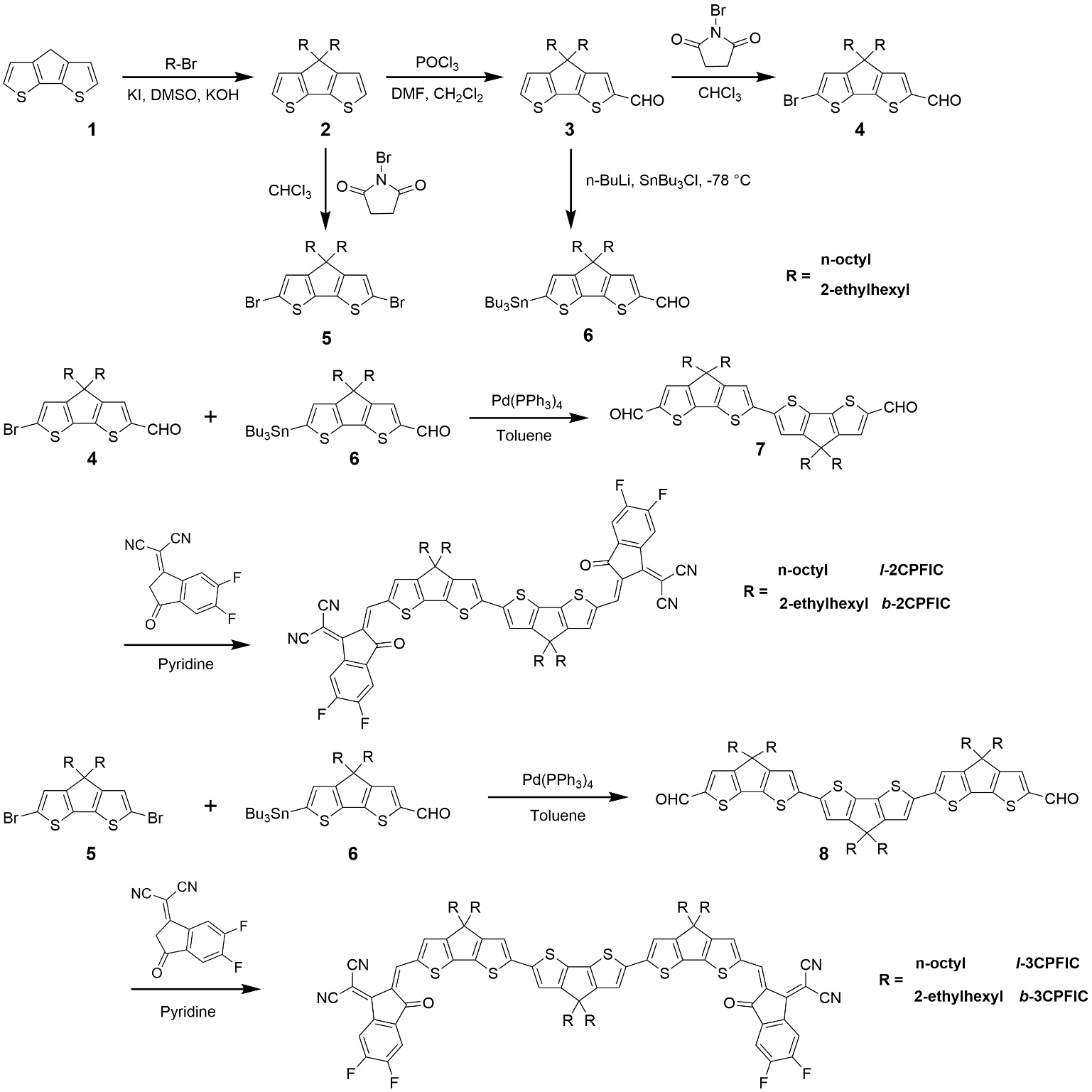


**Scheme S1.** The synthesis routes of *b*-2CPFIC and *b*-3CPFIC.

**Compound 2:** ^1^H NMR (400 MHz, CDCl3) *δ* 7.11 (d, *J* = 4.9 Hz, 2H), 6.93 (dt, *J* = 4.6, 2.1 Hz, 2H), 1.91 – 1.82 (m, 4H), 1.03 – 0.86 (m, 18H), 0.78 – 0.73 (m, 6H), 0.61 – 0.57 (m, 6H).

**Compound 3:** ^1^H NMR (400 MHz, CDCl3) *δ* 9.83 (s, 1H), 7.56 (t, *J* = 3.6 Hz, 1H), 7.38 (d, *J* = 4.9 Hz, 1H), 7.00 (dt, *J* = 4.9, 2.5 Hz, 1H), 2.03 – 1.81 (m, 4H), 1.06 – 0.81 (m, 18H), 0.76 – 0.72 (m, 6H), 0.62 – 0.54 (m, 6H).

**Compound 4:** ^1^H NMR (400 MHz, CDCl3) δ 9.83 (s, 1H), 7.55 (t, *J* = 3.8 Hz, 1H), 7.02 (t, *J* = 3.7 Hz, 1H), 1.93 – 1.77 (m, 5H), 1.07 – 0.85 (m, 18H), 0.80 – 0.72 (m, 6H), 0.64 – 0.59 (m, 6H).

**Compound 5:** ^1^H NMR (400 MHz, CDCl3) δ 6.93 (t, *J* = 3.7 Hz, 2H), 1.90 – 1.71 (m, 4H), 1.07 – 0.87 (m, 18H), 0.78 (t, *J* = 6.9 Hz, 6H), 0.64 – 0.59 (m, 6H).

**Compound 6:** ^1^H NMR (400 MHz, CDCl3) δ 9.84 (d, *J* = 9.6 Hz, 1H), 7.60 – 7.56 (m, 1H), 7.03 – 7.01 (m, 1H), 1.98 – 1.89 (m, 4H), 1.71 – 1.52 (m, 8H), 1.40 – 1.34 (m, 4H), 1.19 – 1.11 (m, 4H), 1.07 – 0.83 (m, 29H), 0.77 (t, *J* = 6.9 Hz, 6H), 0.65 – 0.57 (m, 6H).

**Compound 7:** ^1^H NMR (400 MHz, CDCl3) δ 9.84 (s, 2H), 7.57 – 7.56 (m, 2H), 7.12 – 7.10 (m, 2H), 1.98 – 1.89 (m, 8H), 1.03 – 0.88 (m, 36H), 0.77 – 0.69 (m, 12H), 0.66 – 0.61 (m, 12H).

**Compound 8:** ^1^H NMR (400 MHz, CDCl3) δ 9.82 (s, 2H), 7.56 – 7.54 (m, 2H), 7.13 – 6.98 (m, 4H), 1.96 – 1.86 (m, 12H), 1.05 – 0.90 (m, 48H), 0.77 – 0.61 (m, 42H).

**Compound *b*-2CPFIC:** ^1^H NMR (400 MHz, CDCl3) δ 8.90 (s, 2H), 8.55 – 8.51 (m, 2H), 7.69 – 7.64 (m, 4H), 7.24 (br, 2H), 2.08 – 1.90 (m, 8H), 1.06 – 0.91 (m, 32H), 0.79 – 0.65 (m, 28H). MALDI-TOF-MS: m/z: [M]^+^ calcd for C_76_H_78_F_4_N_4_O_2_S_4_: 1282.494; found: 1282.489.

**Compound *b*-3CPFIC:** ^1^H NMR (400 MHz, CDCl3) δ 8.88 (s, 2H), 8.54 – 8.49 (m, 2H), 7.66 – 7.61 (m, 4H), 7.19 (s, 2H), 7.11 (s, 2H), 2.02 – 1.92 (m, 12H), 1.05 – 0.95 (m, 48H), 0.80 – 0.62 (m, 42H). MALDI-TOF-MS: m/z: [M+H]^+^ calcd for C_101_H_115_F_4_N_4_O_2_S_6_: 1682.720; found: 1682.629.

**Figure S1.** ^1^H NMR spectrum of compound 2 in CDCl_3_.

**Figure S2.** ^1^H NMR spectrum of compound 3 in CDCl_3_.

**Figure S3.** ^1^H NMR spectrum of compound 4 in CDCl_3_.

**Figure S4.** ^1^H NMR spectrum of compound 5 in CDCl_3_.

**Figure S5.** ^1^H NMR spectrum of compound 6 in CDCl_3_.

**Figure S6.** ^1^H NMR spectrum of compound 7 in CDCl_3_.

**Figure S7.** ^1^H NMR spectrum of compound 8 in CDCl_3_.

**Figure S8.** ^1^H NMR spectrum of compound *b*-2CPFIC in CDCl_3_.

**Figure S9.** ^1^H NMR spectrum of compound *b*-3CPFIC in CDCl_3_.


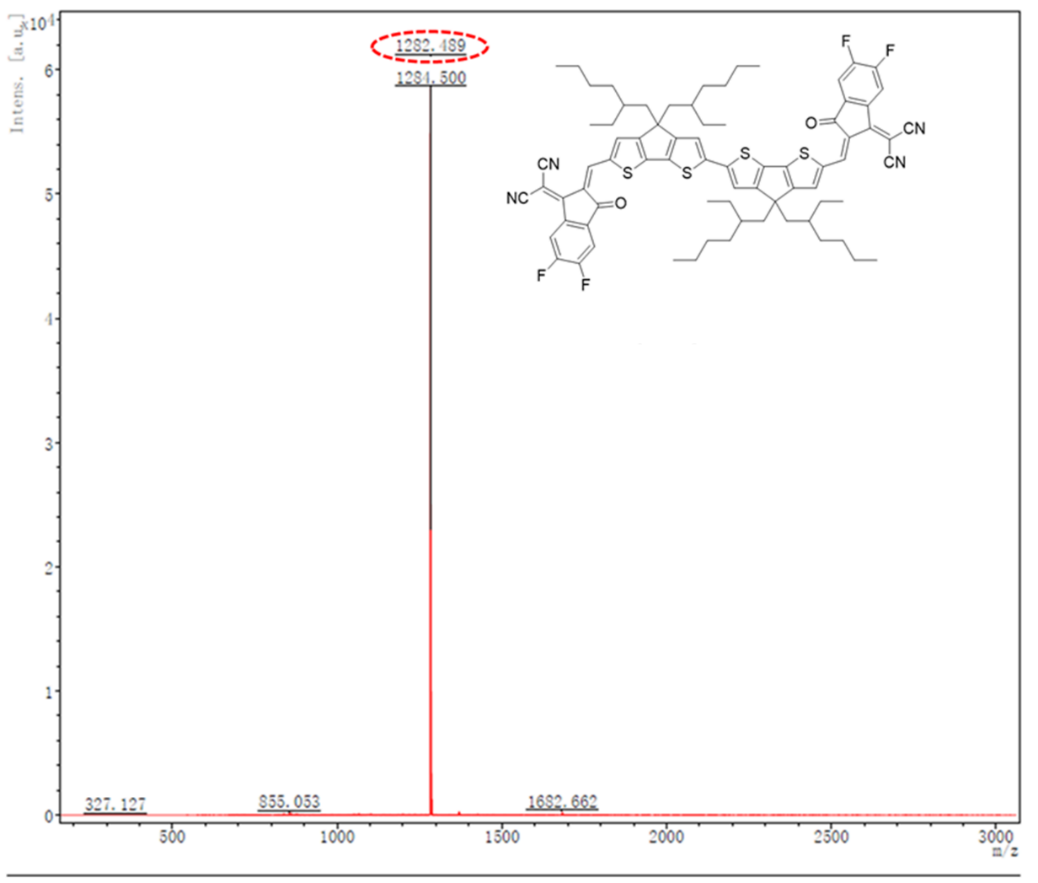


**Figure S10.** High-resolution MALDI-TOF mass spectrum for *b*-2CPFIC.


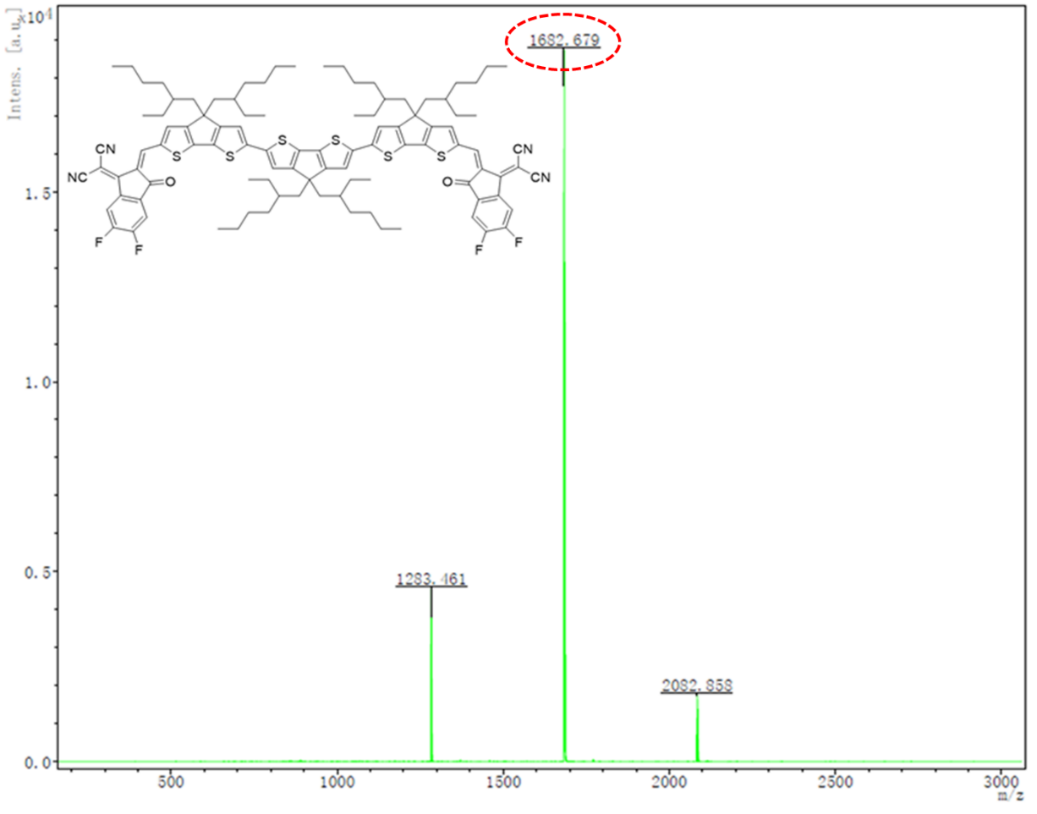


**Figure S11.** High-resolution MALDI-TOF mass spectrum for *b*-3CPFIC.

**Figure S12.** (A) Molar absorption coefficient and (B) normalized photoluminescence (PL) spectra of *l*-2CPFIC, *b*-2CPFIC, *l*-3CPFIC and *b*-3CPFIC in THF solution ( [c]= 10 $\mu$M).


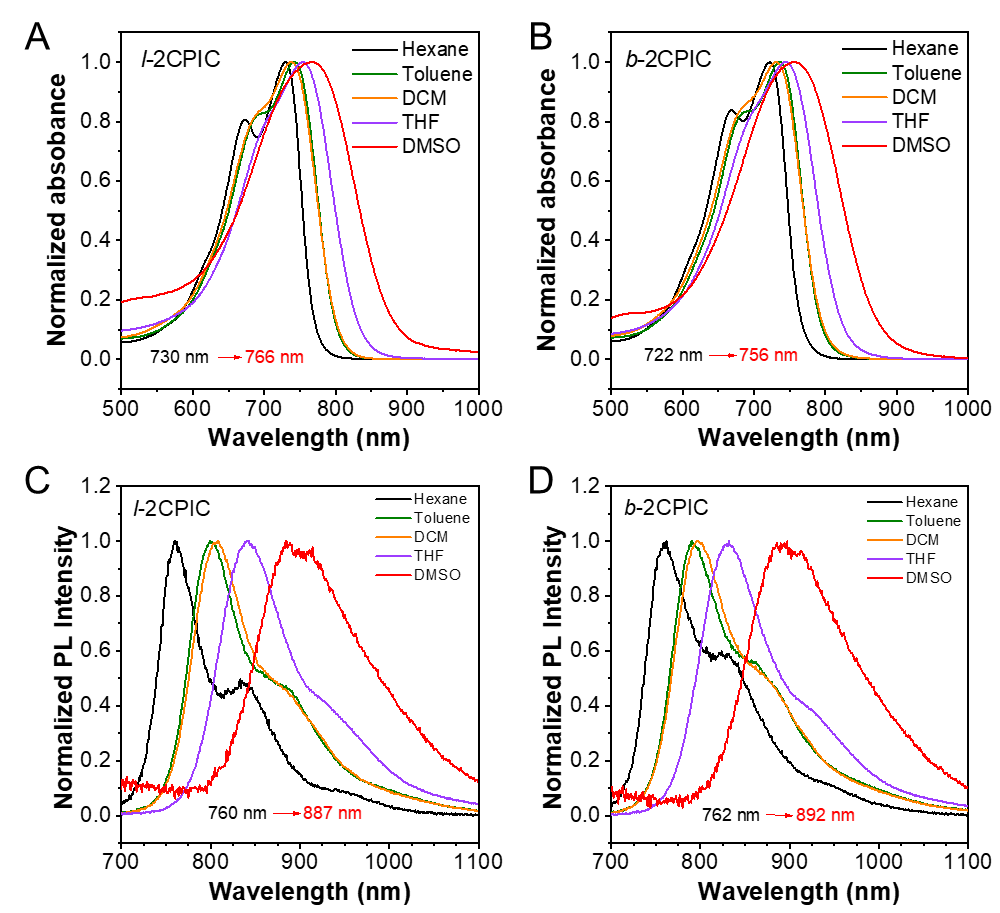


**Figure S13.** Normalized UV-vis-NIR absorption spectra of (A) *l*-2CPFIC and (B) *b*-2CPFIC, and normalized PL spectra of (C) *l*-2CPFIC and (D) *b*-2CPFIC in different solvents.


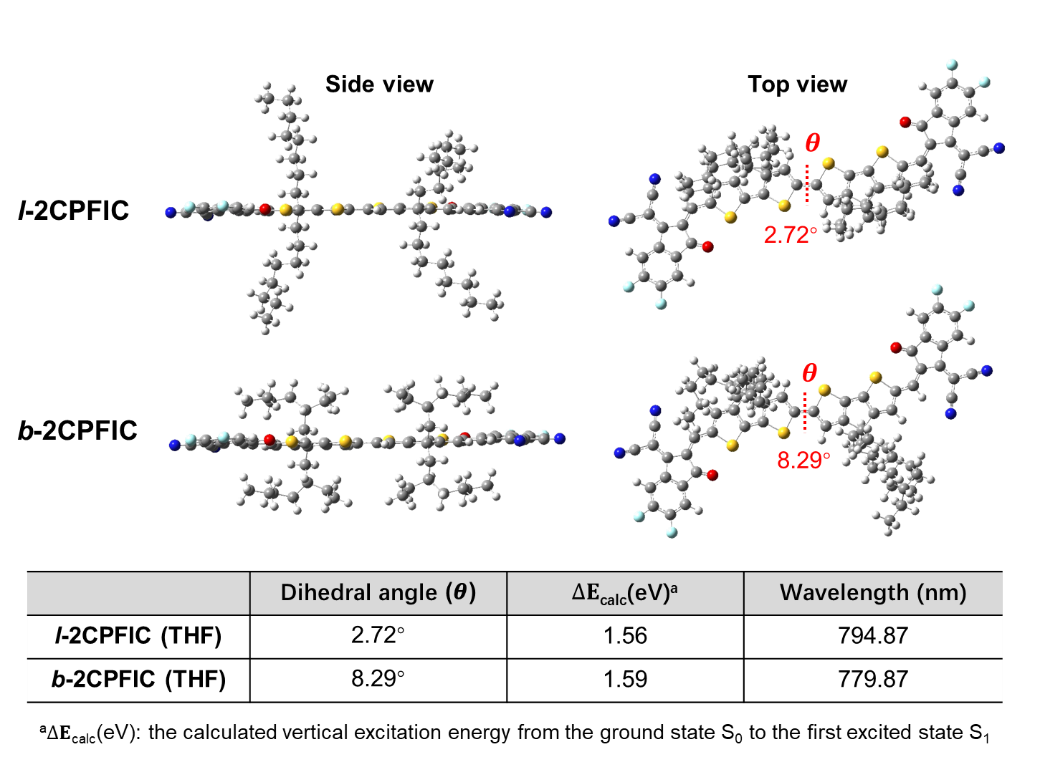


**Figure S14.** Optimized single-molecule conformations and theoretical energy levels of *l*-2CPFIC and *b*-2CPFIC in THF solution. Side views and top views of the molecular backbones optimized under the implicit THF solvent environment. The accompanying table summarizes the corresponding dihedral angles (𝜽), calculated vertical excitation energies ($\Delta E$_calc_), and simulated absorption wavelengths.

**Figure S15.** Illustrations of the frontier molecular orbitals (HOMOs and LUMOs) of molecular fluorophores 2CPFIC and 3CPFIC at the B3LYP-D3/def2-SVP level (isovalue = 0.05 a.u.).

**Table S1**. Calculated packing density and fractional free volume (FFV) for the aggregates of *l*-2CPFIC and *b*-2CPFIC.

|  | **Packing density (kg/m^3^)** | **Free volume fraction** |
| --- | --- | --- |
| ***l*-2CPFIC** | 899.422 | 53.29% |
| ***b*-2CPFIC** | 1076.392 | 43.62% |


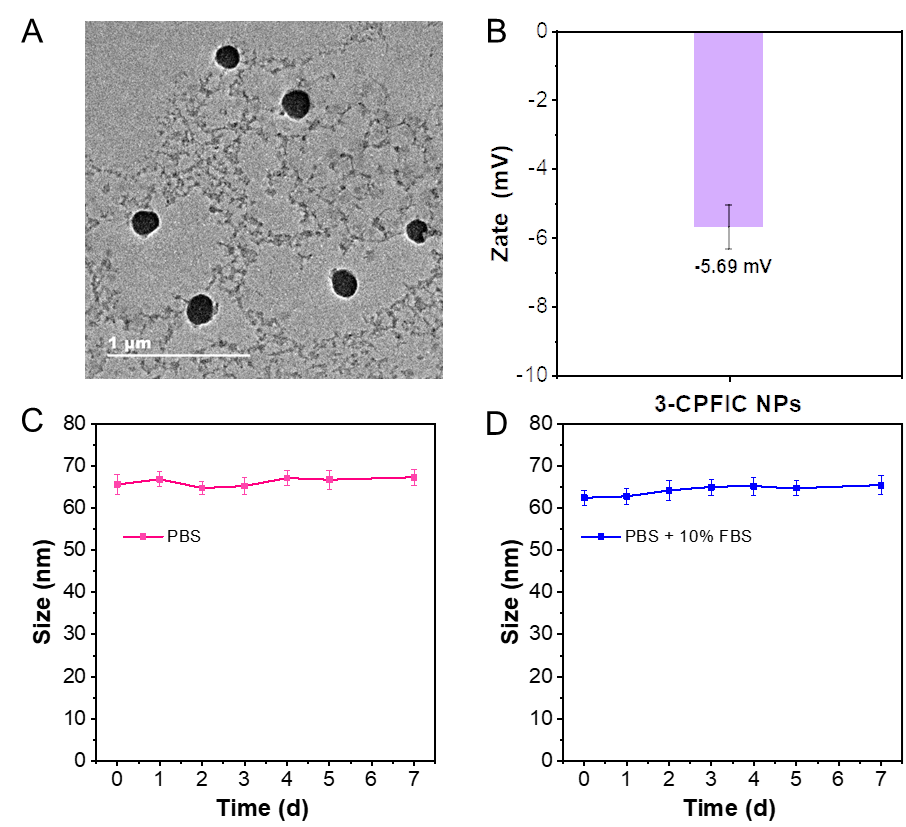


**Figure S16.** (A) TEM image of b-3CPFIC NPs in an aqueous solution. (B) Zeta potential of b-3CPFIC NPs in water (n = 3). (C, D) Size variations of b-3CPFIC NPs in PBS solution and PBS + 10% FBS within 7 days (n = 3).


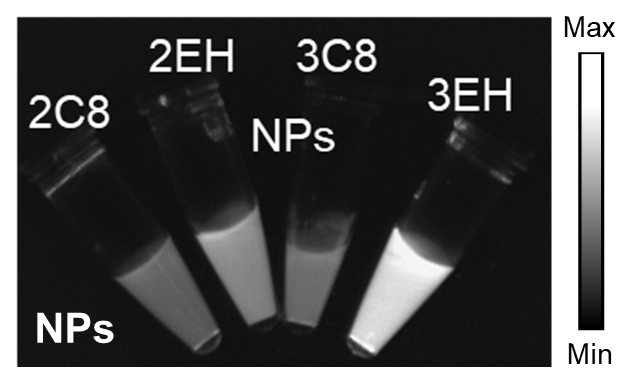


**Figure S17.** NIR-II fluorescence imaging of different NPs with concentration of 10 µM under 808 nm laser irradiation using 1000 nm LP filter.

**Figure S18.** Data in NIR-II QY calculation. PL spectra of different samples with OD values at 808 nm around 0.1, 0.08, 0.06, 0.04, and 0.02: (A) IR26 in dichloroethane, (B) *l*-2CPFIC NPs, (C) *b*-2CPFIC NPs, (D) *l*-3CPFIC NPs and (E) *b*-3CPFIC NPs in ultrapure water. (F) Linear relationship between integrated fluorescence intensity (850 ~ 1400 nm) and absorbance value at 808 nm of these NPs and IR26.


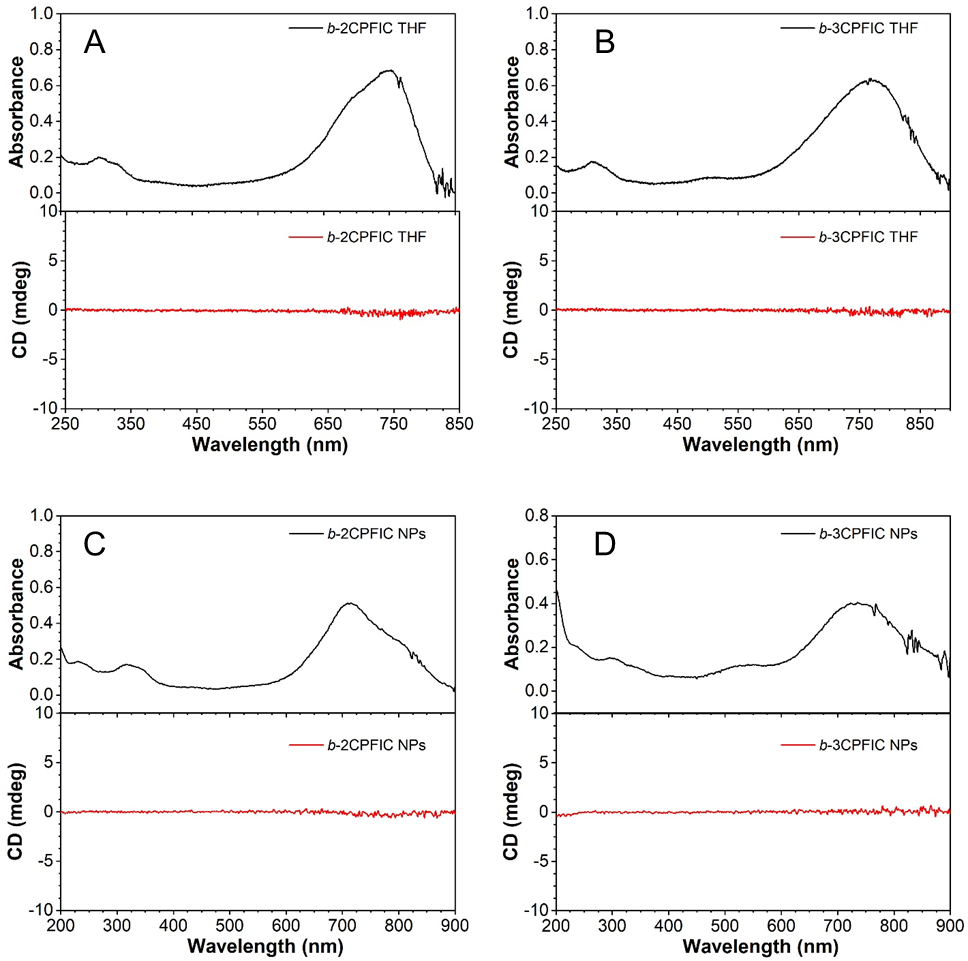


**Figure S19.** CD spectra of *b*-2CPFIC and *b*-3CPFIC in solution state and nanoparticle state.

**Figure S20.** The fluorescence spectra changes of DCFH for ROS detection. (A) alone DCFH, (B) *l*-2CPFIC NPs, (C) *b*-2CPFIC NPs, (D) *l*-3CPFIC NPs and (E) *b*-3CPFIC NPs with increasing irradiation time under an 808 nm laser (0.5 W cm^-2^).


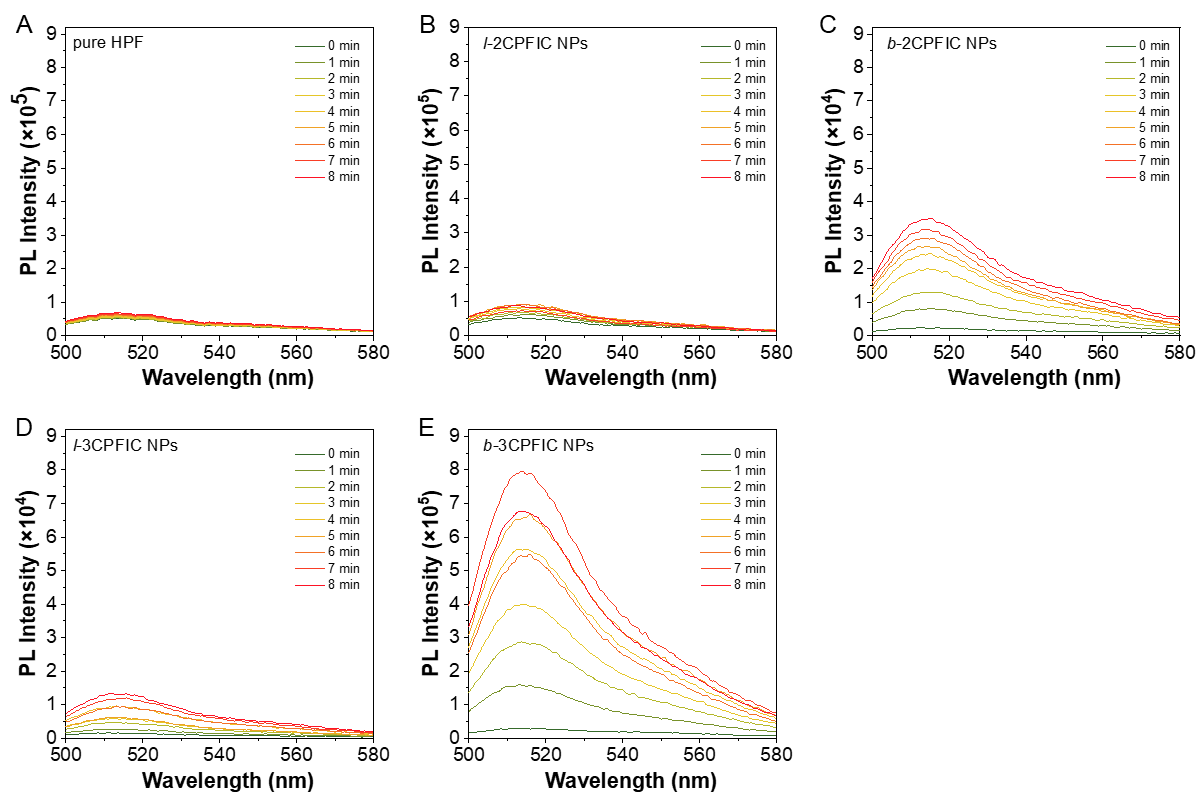


**Figure S21.** PL spectra of HPF in the presence of (A) blank, (B) *l*-2CPFIC NPs, (C) *b*-2CPFIC NPs, (D) *l*-3CPFIC NPs and (E) *b*-3CPFIC NPs under 808 nm irradiation (0.5 W cm^-2^). [PSs] = 10 μM, [HPF] = 20 μM.


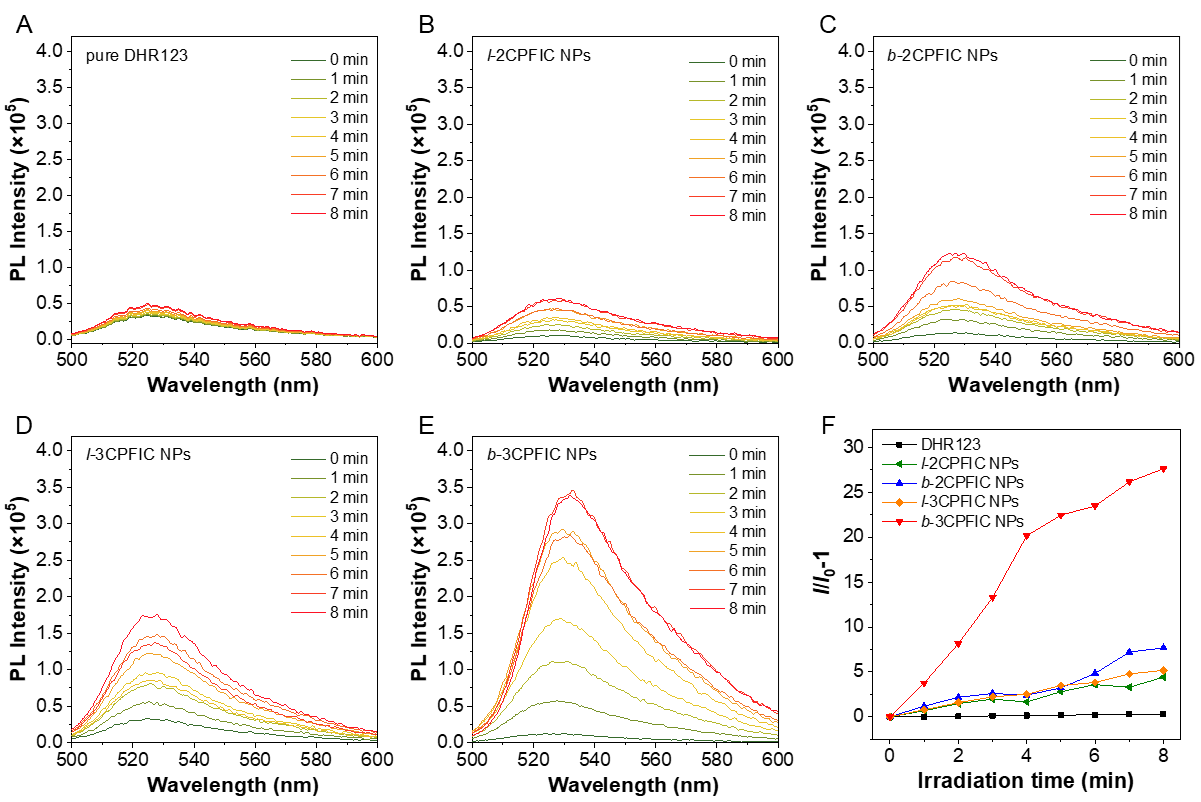


**Figure S22.** PL spectra of DHR 123 in the presence of (A) blank, (B) *l*-2CPFIC NPs, (C) *b*-2CPFIC NPs, (D) *l*-3CPFIC NPs and (E) *b*-3CPFIC NPs under 808 nm irradiation (0.5 W cm^-2^). [PSs] = 10 μM, [DHR 123] = 20 μM.


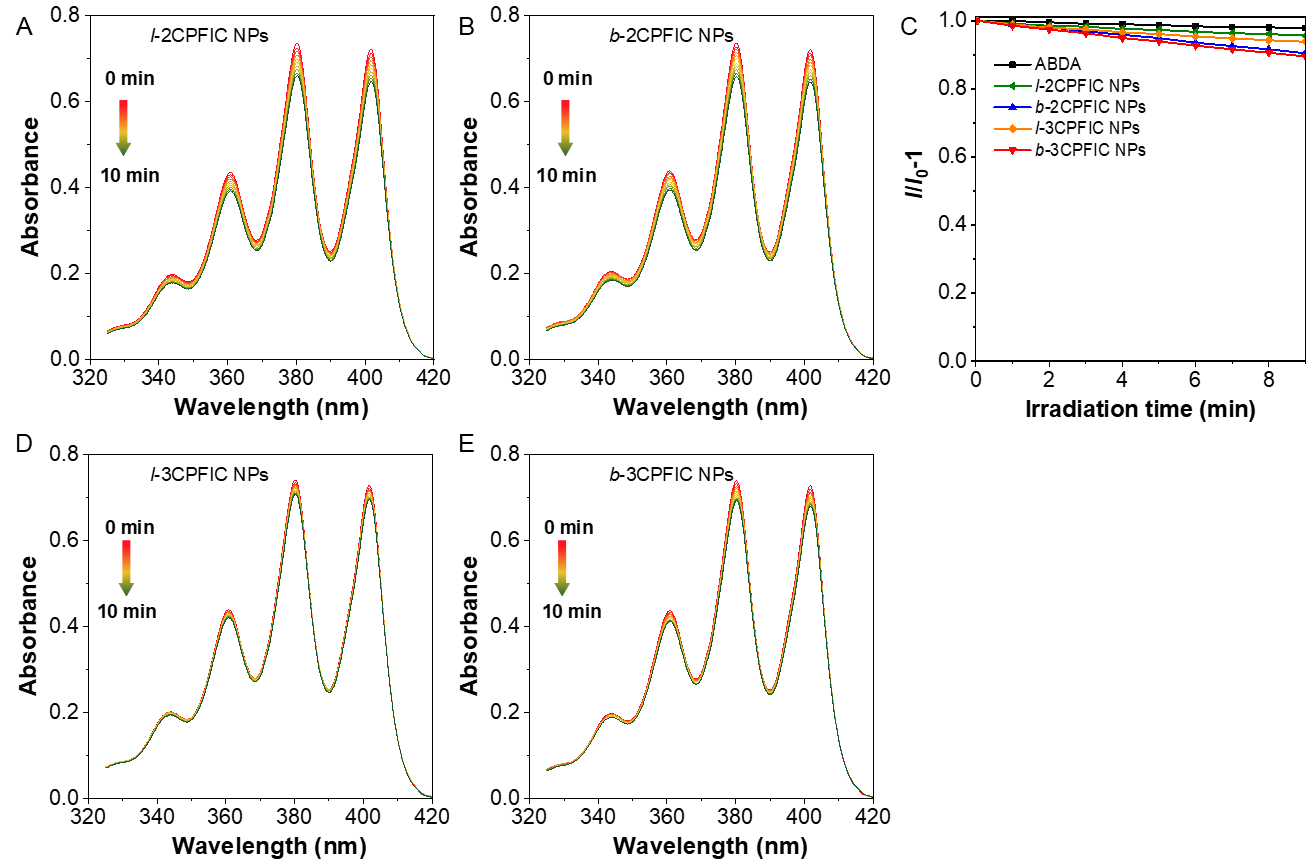


**Figure S23.** The absorbance spectra of ABDA in the presence of (A) *l*-2CPFIC NPs, (B) *b*-2CPFIC NPs, (D) *l*-3CPFIC NPs and (E) *b*-3CPFIC NPs under 808 nm irradiation (0.5 W cm^-2^) and decomposition rate of ABDA (C) under 808 nm irradiation (0.5 W cm^-2^). [PSs] = 10 μM, [ABDA] = 20 μM.


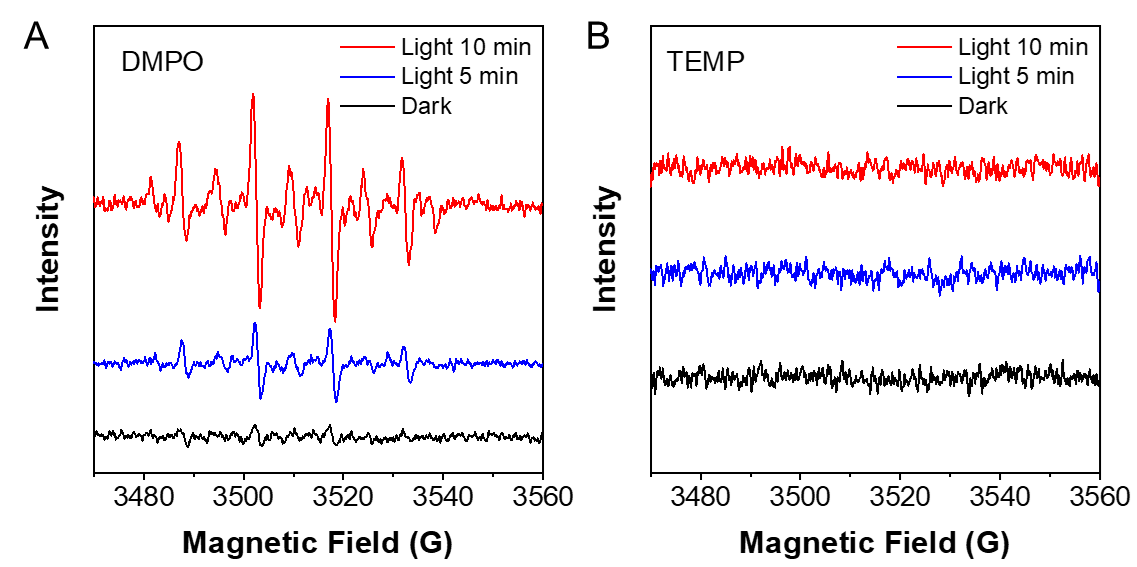


**Figure S24.** EPR spectra of ROS generated by *b*-3CPFIC NPs under light irradiation using (A) 5,5-dimethyl-1-pyrroline N-oxide (DMPO) and (B) 2,2,6,6-Tetramethylpiperidine (TEMP) as spin-trapping.





**Figure S25.** The photothermal conversation efficiency of four NPs. (A) *l*-2CPFIC NPs, (B) *b*-2CPFIC NPs, (C) *l*-3CPFIC NPs and (D) *b*-3CPFIC NPs.

**Figure S26.** Optimized ground-state (S_0_) (left) and first singlet excited state (S_1_) (right) geometries of 2CPFIC and 3CPFIC at the optimally the B3LYP/TZVP level. The dihedral angels are also listed.


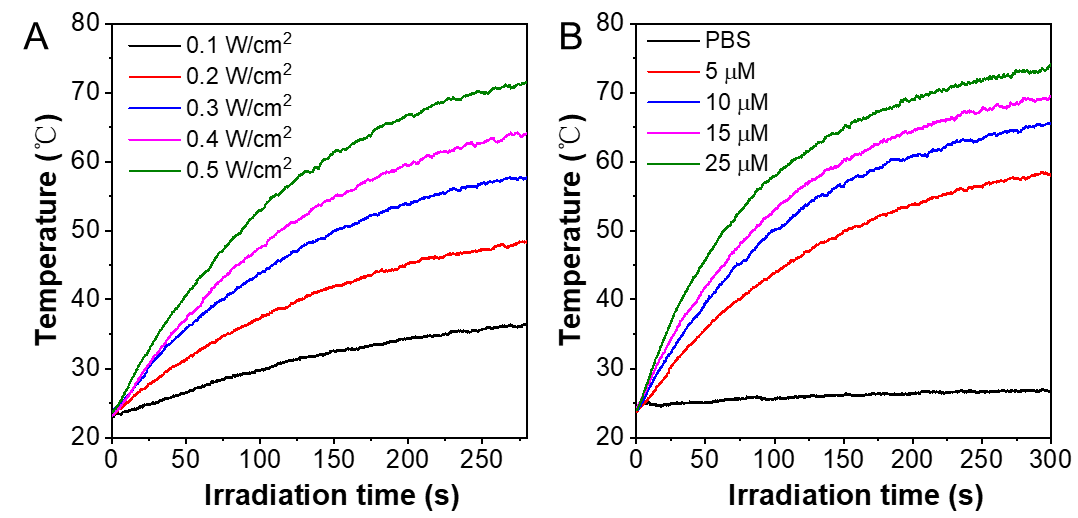


**Figure S27.** (A) The temperature variation of *b*-3CPFIC NPs (10 μM) at different optical density intensities under 808 nm laser irradiation. (B) The photothermal response of *b*-3CPFIC NPs at different concentrations under 808 nm laser irradiation (0.3 W cm^-2^).


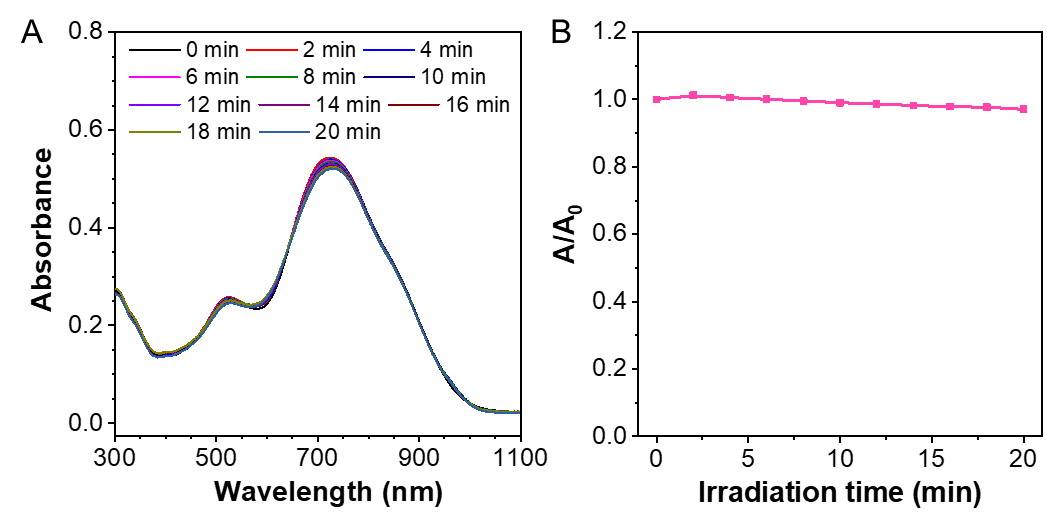


**Figure S28.** (A) Absorption spectra and (B) relative absorbance change (*A*/*A*_0_) of *b*-3CPFIC NPs under continuous irradiation for different times (808 nm laser, 0.5 W cm^-2^).


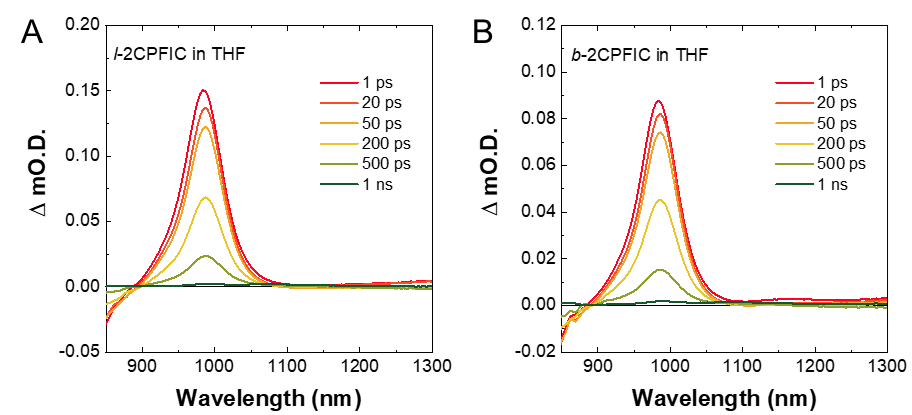


**Figure S29.** The TA spectra of *l*-2CPFIC and *b*-2CPFIC molecule state (in THF) probed at the 850-1300 nm region at different time delays following photoexcitation at 720 nm.


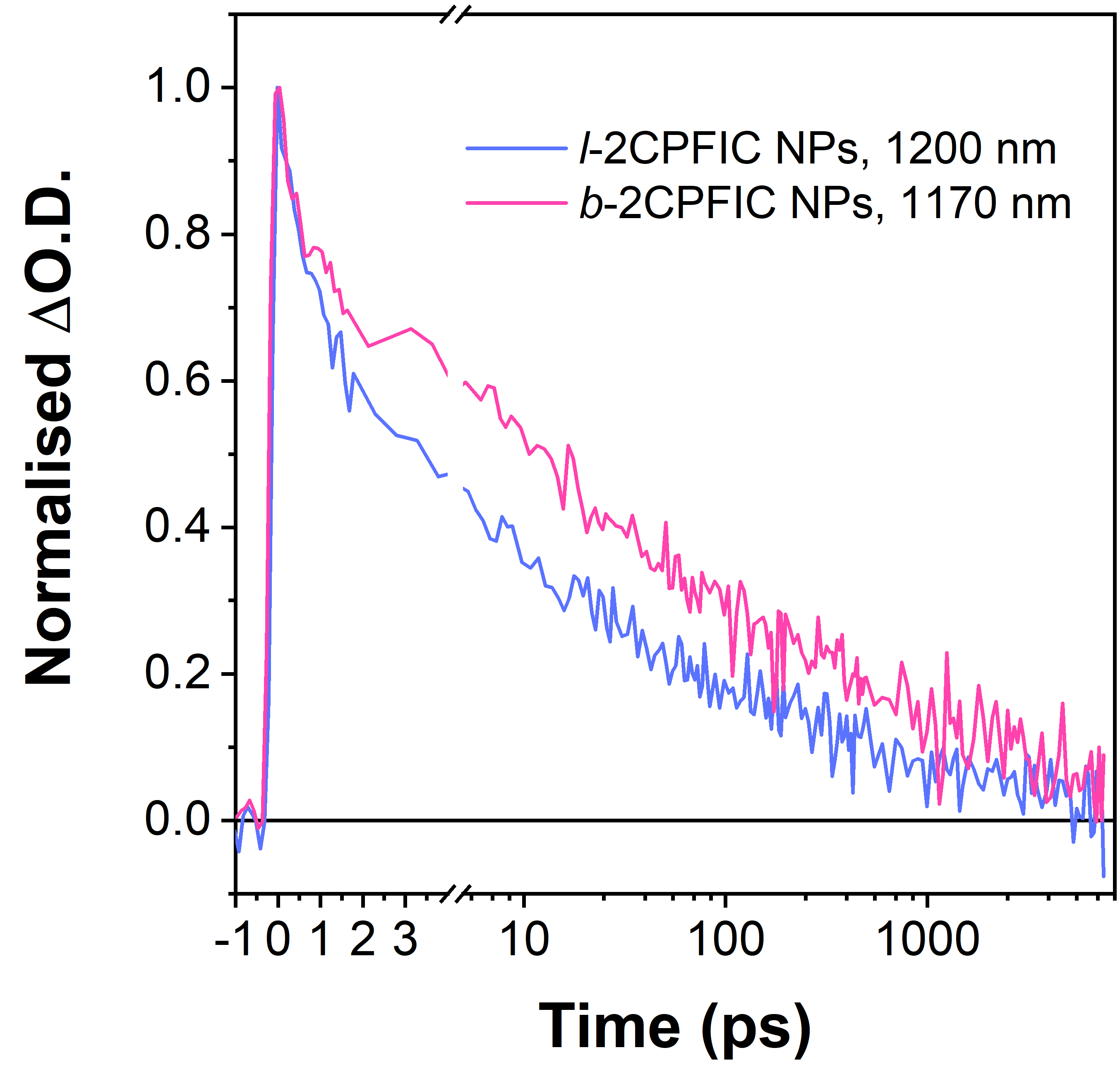


**Figure S30.** The kinetic traces probed at selected ESA of *l*-2CPFIC NPs and *b*-2CPFIC NPs.


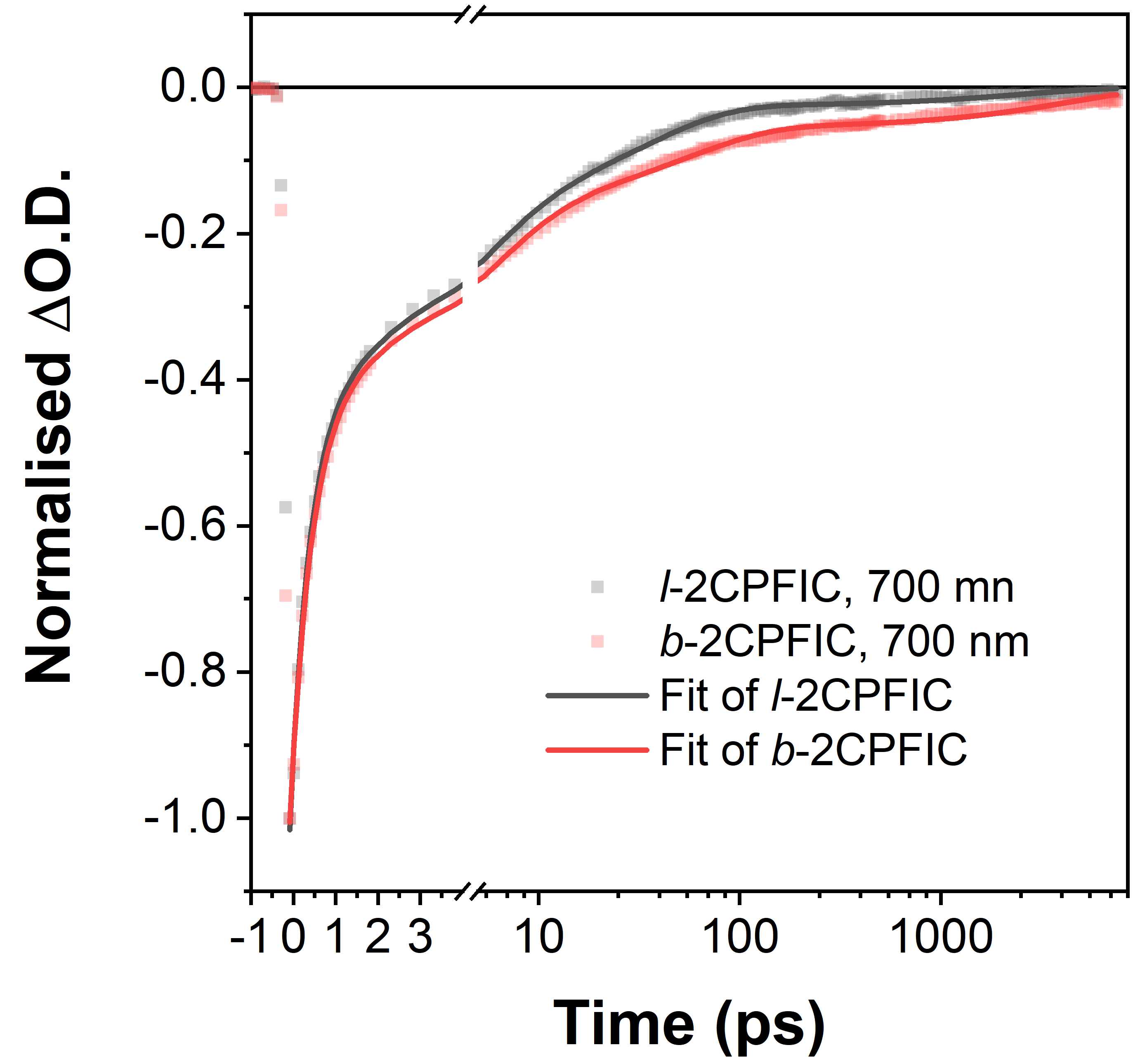


**Figure S31.** The kinetic traces and fitting curves of *l*-2CPFIC and *b*-2CPFIC NPs probed at GSB.

**Table S2.** Fitting parameters of ground-state bleaching (GSB) around the maximum absorption of samples.

| Sample | $\boldsymbol{\tau}$**_1_ (ps)/*A*_1_**  **(%)** | $\boldsymbol{\tau}$**_2_ (ps)/*A*_2_**  **(%)** | $\boldsymbol{\tau}$**_3_ (ps)/*A*_3_**  **(%)** | $\boldsymbol{\tau}$**_4_ (ps)/*A*_4_**  **(%)** |
| --- | --- | --- | --- | --- |
| *l*-2CPFIC NPs | 0.44 ± 0.01 (49.2%) | 4.2 ± 0.2 (31.2%) | 33.0 ± 1.7 (16.8%) | 2580.8 ± 293.3 (2.8%) |
| *b*-2CPFIC NPs | 0.49 ± 0.01 (50.2%) | 5.0 ± 0.2 (30.8%) | 53.0 ± 2.8 (13.0%) | 4380.6 ± 242.3 (6.0%) |
| *l*-3CPFIC NPs | 0.71 ± 0.02 (41.5%) | 8.8 ± 0.3 (35.6%) | 46.4 ± 2.3  (16.4%) | 5613.3 ± 327.2 (6.5%) |
| *b*-3CPFIC NPs | 0.52 ± 0.01 (46.4%) | 5.1 ± 0.2 (32.7%) | 75.3 ± 3.8  (15.0%) | 7338.9 ± 482.8 (5.9%) |

*A* denote the fraction of excited population of the associated component.


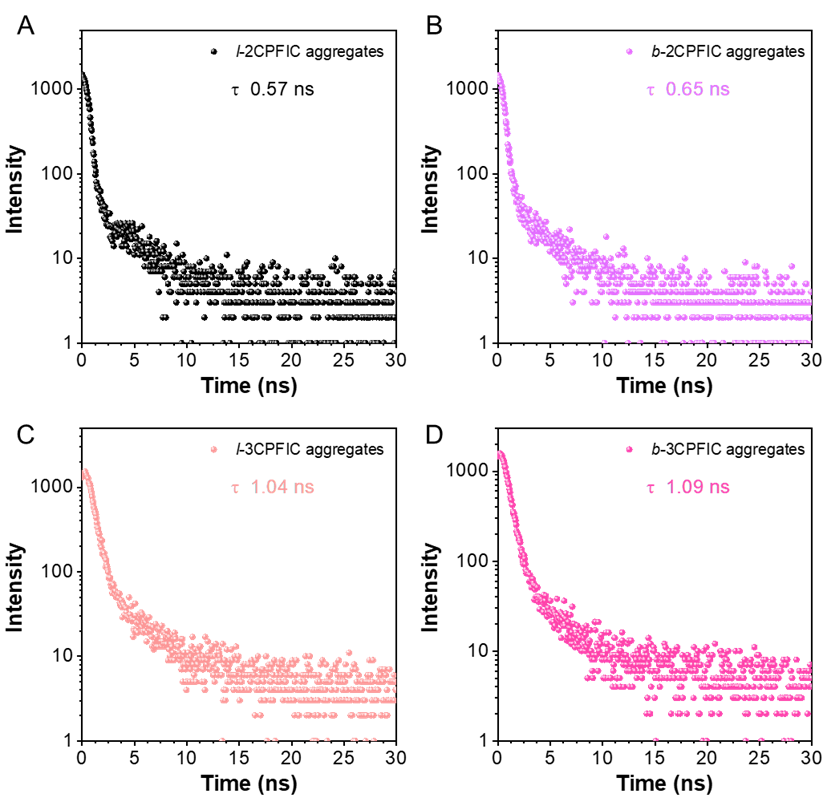


**Figure S32.** Transient fluorescence decay curves of *l*-2CPFIC, *b*-2CPFIC, *l*-3CPFIC and *b*-3CPFIC in 95% Hexane/CHCl_3._


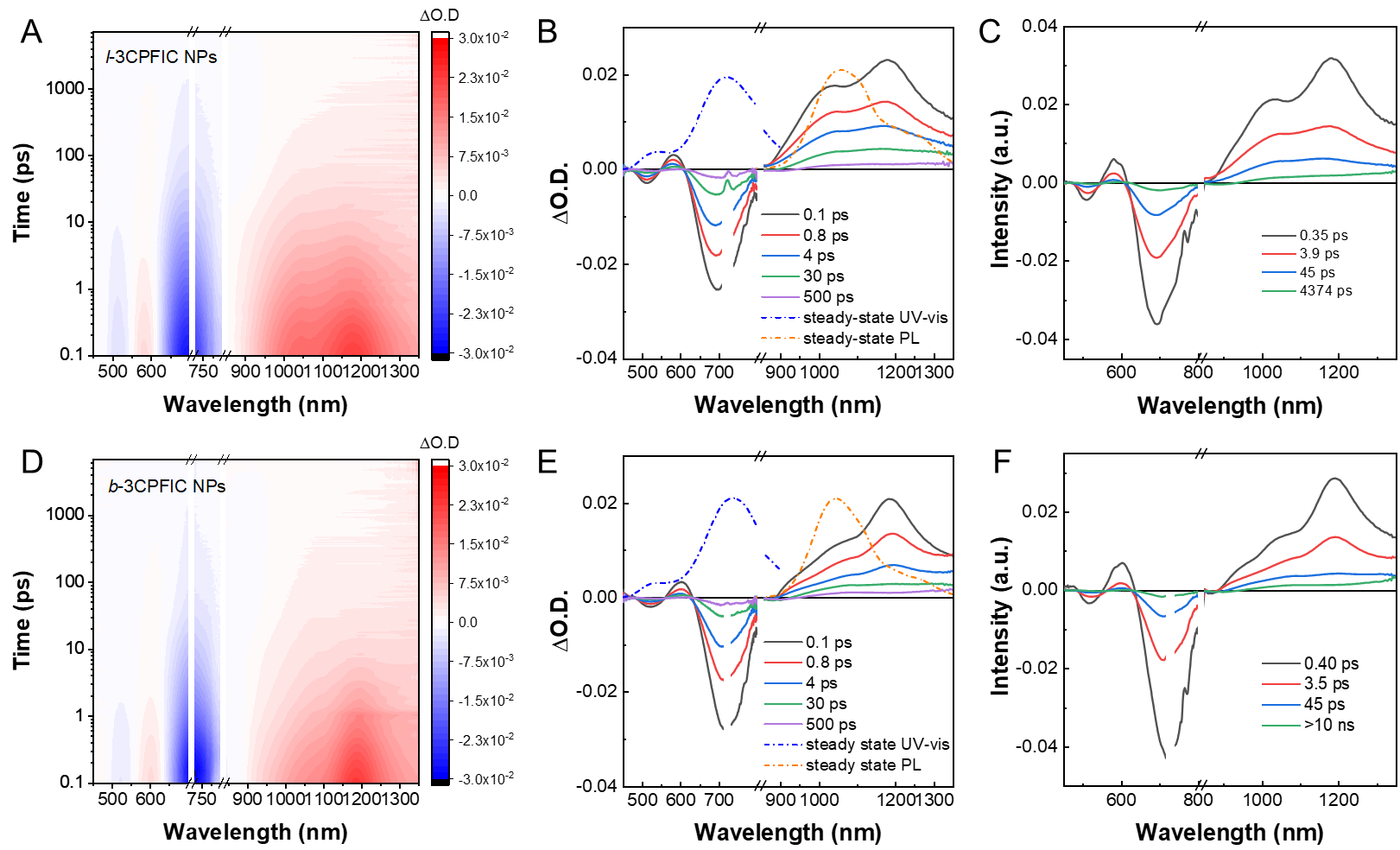


**Figure S33.** Raw data and global analysis of fs-TAS data upon 720 nm excitation. 2D color femtosecond transient absorption (TA) mapping of (A) *l*-3CPFIC NPs and (D) *b*-3CPFIC NPs in water following photoexcitation with 720 nm laser plus. The fs-TA spectra of (B) *l*-3CPFIC NPs and (E) *b*-3CPFIC NPs at different pump-probe time delays. The corresponding evolution associated spectra (EAS) obtained from global lifetime analysis for (C) *l*-3CPFIC NPs and (F) *b*-3CPFIC NPs.


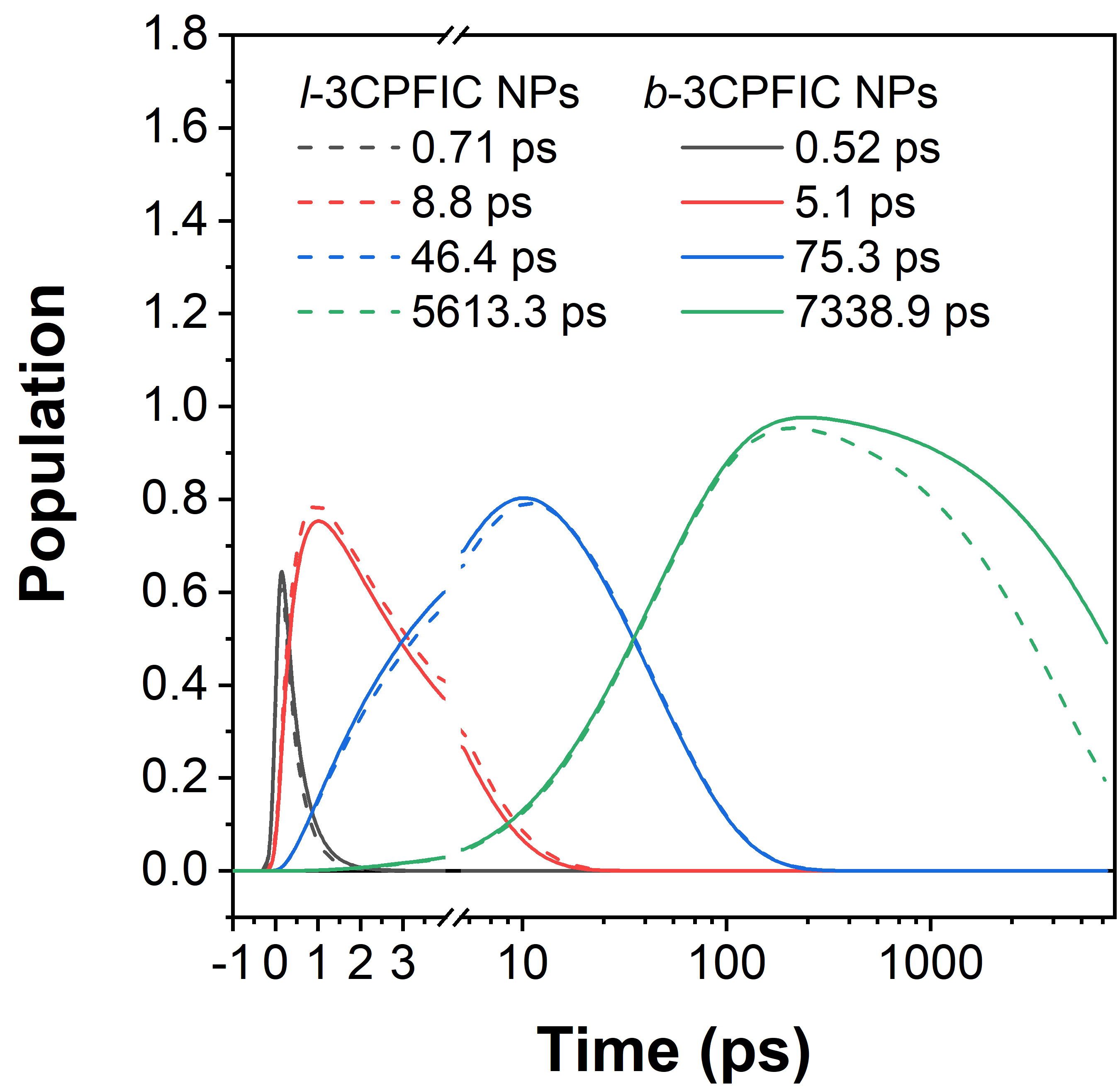


**Figure S34.** The calculated amplitudes of the individual species with time obtained from GLA analysis. The dashed lines represent *l*-3CPFIC NPs and the solid lines *b*-3CPFIC NPs.

Table S3. Crystallographic and structural refinement data of *l*-2CPFIC and *b*-2CPFIC.

| Name | *l*-2CPFIC | *b*-2CPFIC |
| --- | --- | --- |
| Empirical formula | C_76_H_78_F_4_N_4_O_2_S_4_ | C_76_H_78_F_4_N_4_O_2_S_4_ |
| Formula weight | 1283.66 | 1283.66 |
| Temperature (K) | 170.00(10) | 218.00 |
| Crystal system | monoclinic | trigonal |
| Space group | P2_1_/c | R-3 |
| a (Å) | 15.6919(2) | 83.661(9) |
| b (Å) | 9.45930(10) | 83.661(9) |
| c (Å) | 26.8917(4) | 14.076(2) |
| α (°) | 90 | 90 |
| β (°) | 97.1350(10) | 90 |
| γ (°) | 90 | 120 |
| Volume (Å^3^) | 3960.74(9) | 85321(23) |
| Z | 2 | 36 |
| *ρ*_calc_g/cm^3^ | 1.076 | 0.899 |
| μ/mm^‑1^ | 1.518 | 0.830 |
| F (000) | 1356.0 | 24408.0 |
| Crystal size/mm^3^ | 0.15 × 0.12 × 0.1 | 0.13 × 0.12 × 0.1 |
| Radiation | Cu Kα (λ = 1.54184) | GaKα (λ = 1.34139) |
| 2*Θ* range for data collection/° | 5.676 to 133.19 | 5.514 to 104.188 |
| Index ranges | -18 ≤ h ≤ 16, -7 ≤ k ≤ 11, -32 ≤ l ≤ 28 | -98 ≤ h ≤ 98, -98 ≤ k ≤ 98, -16 ≤ l ≤ 16 |
| Reflections collected | 19235 | 255454 |
| Independent reflections | 6985 [R_int_ = 0.0391, R_sigma_ = 0.0478] | 32189 [R_int_ = 0.0910, R_sigma_ = 0.0735] |
| Data/restraints/parameters | 6985/363/368 | 32189/1348/1588 |
| Goodness-of-fit on F^2^ | 1.755 | 1.169 |
| Final R indexes [I >= 2σ (I)] | R_1_ = 0.1216, wR_2_ = 0.3771 | R_1_ = 0.1326, wR_2_ = 0.2993 |
| Final R indexes [all data] | R_1_ = 0.1328, wR_2_ = 0.3958 | R_1_ = 0.1958, wR_2_ = 0.3293 |
| Largest diff. peak/hole / e Å^-3^ | 1.84/-1.26 | 0.45/-0.44 |
| CCDC Number | 2404889 | 2404891 |

**Figure S35.** Interlayer dimer structure of *b*-2CPFIC and *l*-2CPFIC.


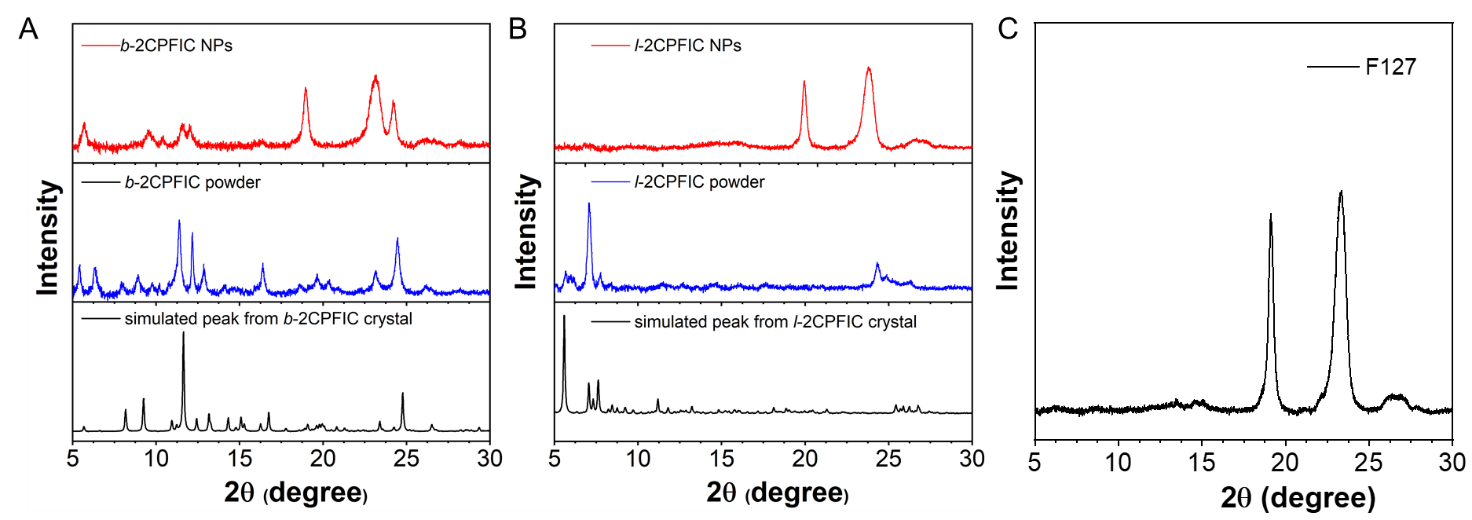


**Figure S36.** XRD patterns of (A) *l*-2CPFIC and (B) *b*-2CPFIC in NPs and powder state comparison with the theoretical powder patterns generated from single-crystal data. All NPs sample were prepared by freeze-drying method and all powdered samples were obtained directly via rotary evaporation. (C) The XRD pattern of F127 polymer obtained through freeze-drying method.


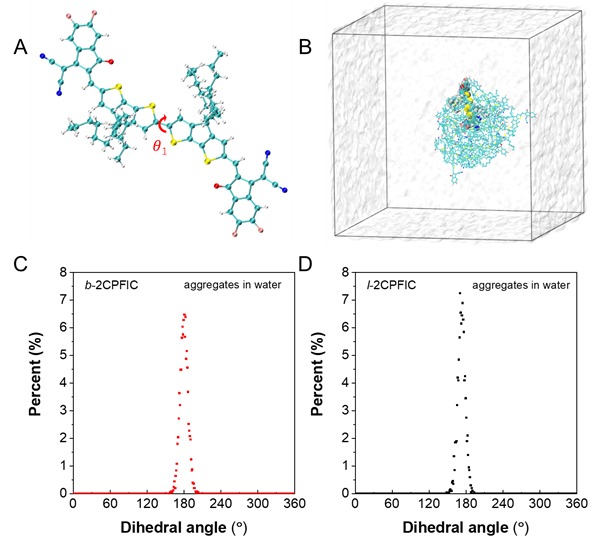


**Figure S37.** (A) Molecular conformation of *b*-2CPFIC. $\theta$1 indicates the dominant dihedral angle between the two CP units. (b) Molecular dynamics simulation snapshot of *b*-2CPFIC aggregates in water. The innermost molecule was shown in space-filling style, while the other molecules were shown in ball-and-stick style. Distributions of the representative dihedral angle$\theta$1 of the innermost molecules in (C) *b*-2CPFIC and (D) *l*-2CPFIC aggregates in water. Compared to the dihedral angles in their corresponding crystals (*b*-2CPFIC: 0°; *l*-2CPFIC: 17.86°), both show remarkable consistency.


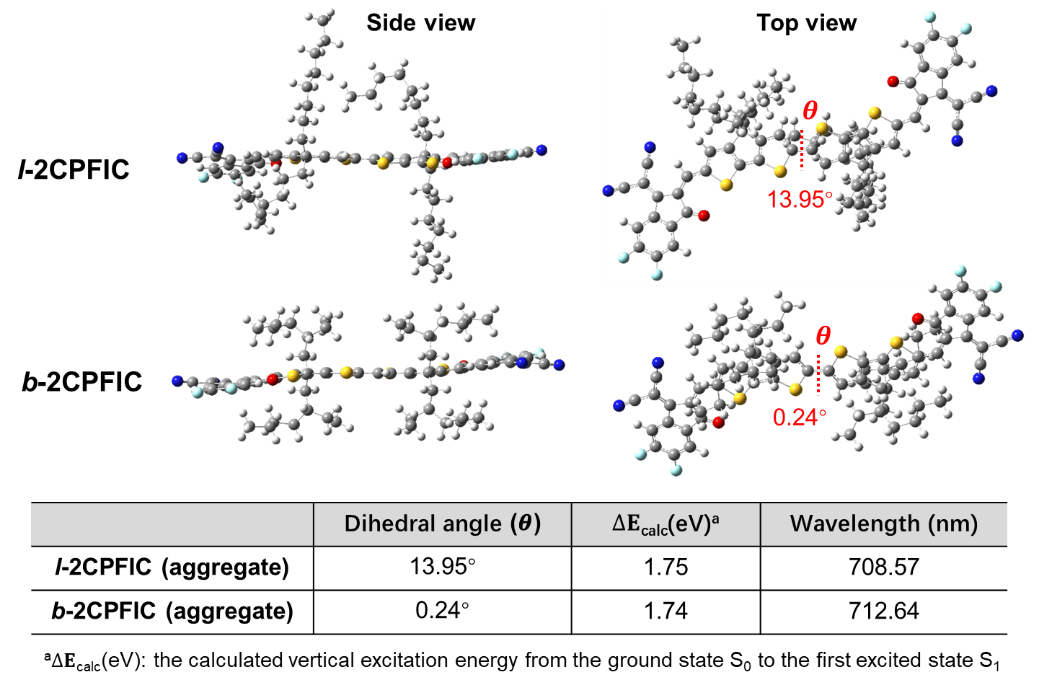


**Figure S38**. Optimized aggregate-state conformations and theoretical energy levels of *l*-2CPFIC and *b*-2CPFIC. Side views and top views of the molecular backbones extracted from the QM/MM optimized crystalline clusters. The accompanying table summarizes the corresponding dihedral angles (𝜽), calculated vertical excitation energies (∆Ecalc), and simulated absorption wavelengths.


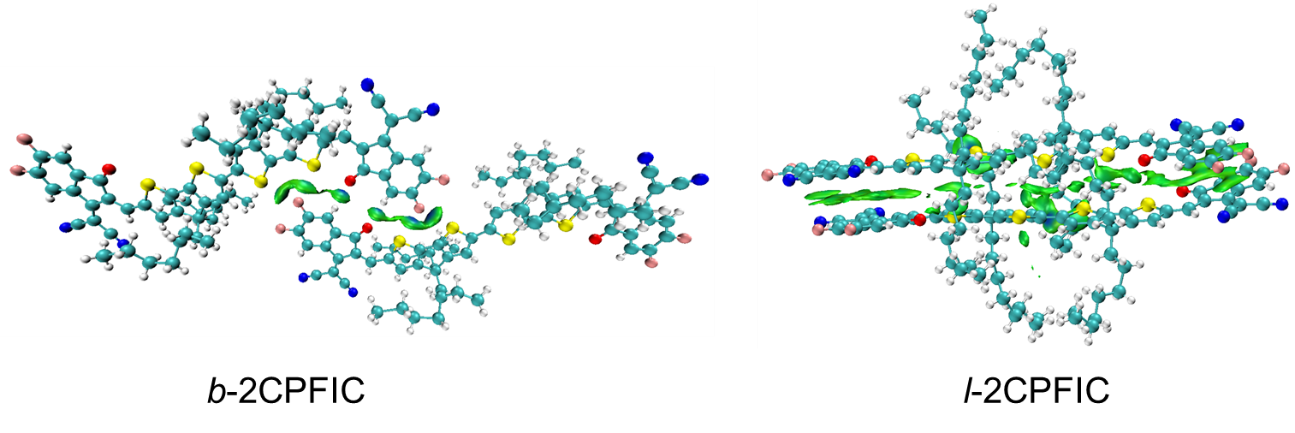


**Figure S39.** Visualized Independent Gradient Model (IGM) analysis of the *b*-2CPFIC and *l*-2CPFIC dimers.


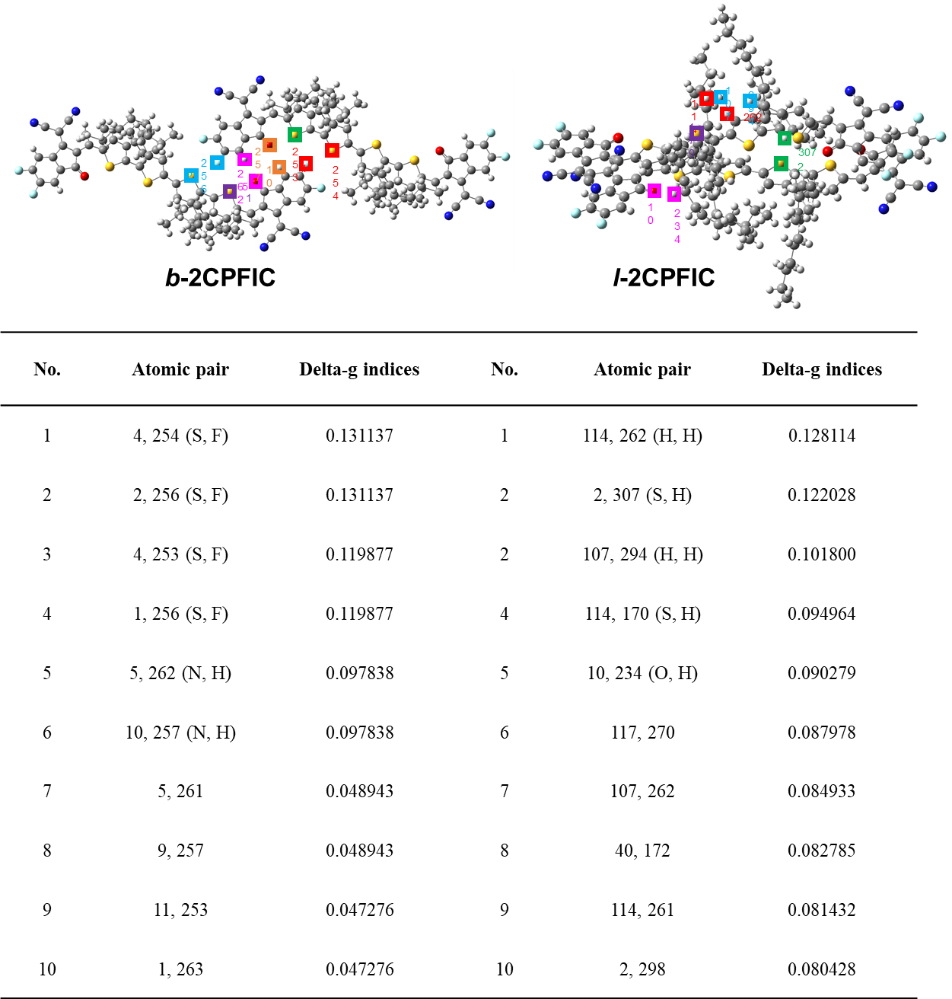


**Figure S40.** Quantitative atomic pair contribution analysis based on the IGM method for *b*-2CPFIC and *l*-2CPFIC dimers.


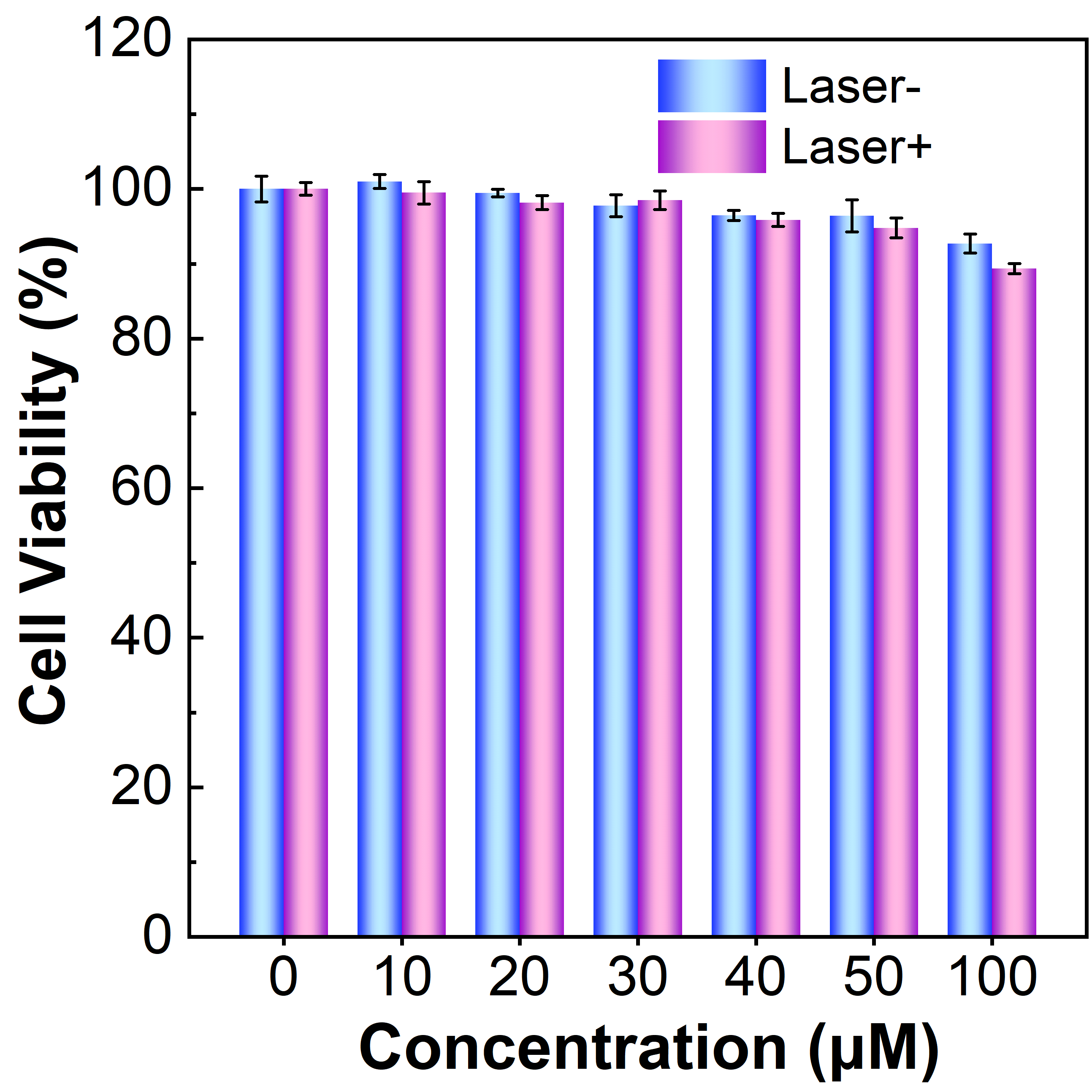


**Figure S41.** Cell viability of NIH-3T3 normal cells after incubation with *b*-3CPFIC NPs of various concentrations with or without 808 nm laser irradiation (0.8 W cm^-2^).


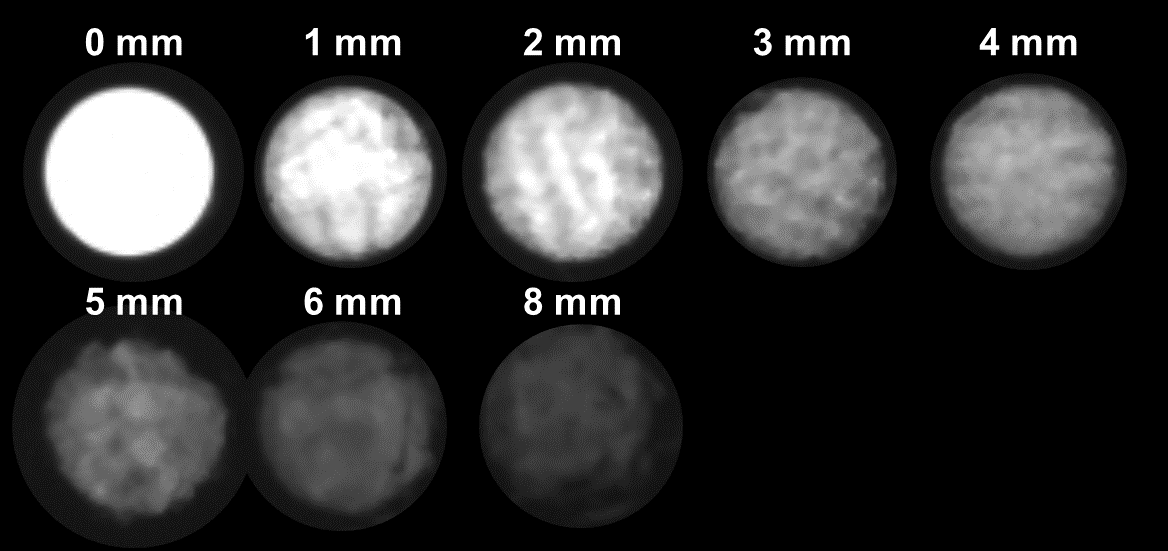


**Figure S42.** Fluorescence images of *b*-3CPFIC NPs covered with tissue-mimicking phantoms with varying thicknesses.

**Figure S43.** (A) NIR-II fluorescence imaging of blood vessels using *b*-3CPFIC NPs under different LP filters and (B) their corresponding cross-sectional intensity profile and signal to noise ratio along the red-dashed line, respectively.


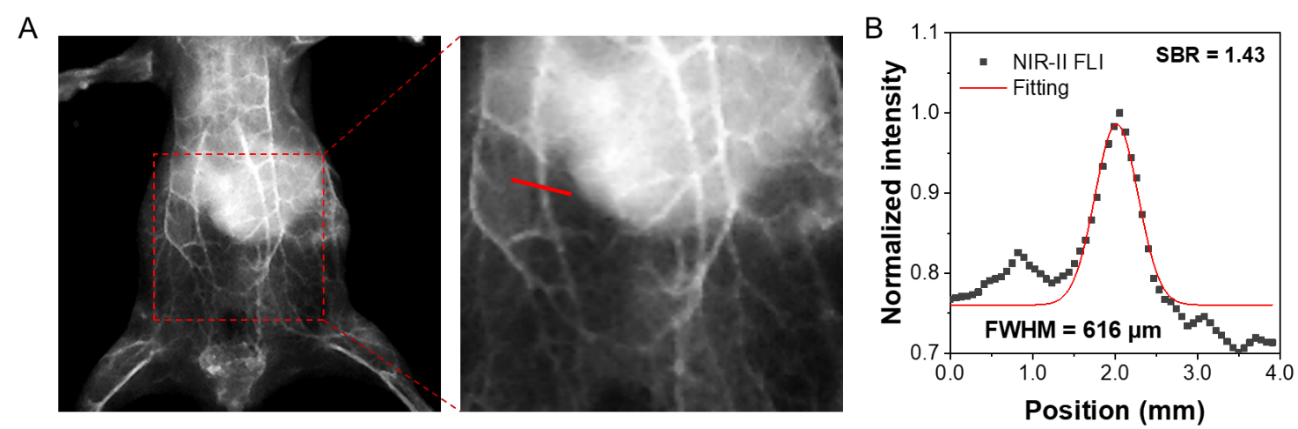


**Figure S44.** (A) The NIR-II imaging of blood vessel of mice in supine position 5 min after intravenous injection of *l*-3CPFIC NPs (100 $\mu$L, 100 $\mu$M) (B) Cross-sectional intensity profile along the red line in Figure A with the peak fitted to a Gaussian function (red curve).

**Figure S45.** (A) The images of the major organs (heart, liver, spleen, lung, kidney) in the NIR-II channel after 2 h post-injection of *b*-3CPFIC NPs. (B) The *I*/*I*_H_ ratio of the major organ (heart, liver, spleen, lung, kidney) after 2 h post-injection of *b*-3CPFIC NPs. *I* represent the PL intensity of the major organ, while *I_H_* indicates the PL intensity of the heart.


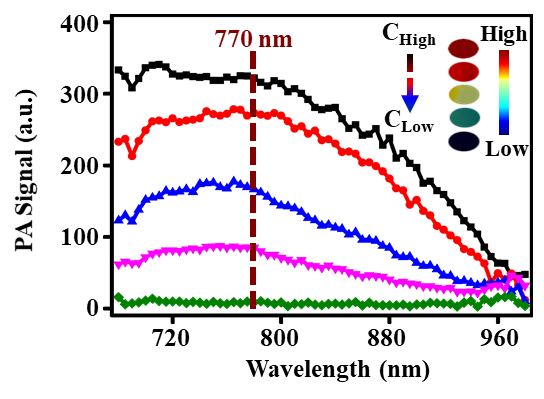


**Figure S46.** The photoacoustic (PA) signal intensity of *b*-3CPFIC NPs with different concentration.


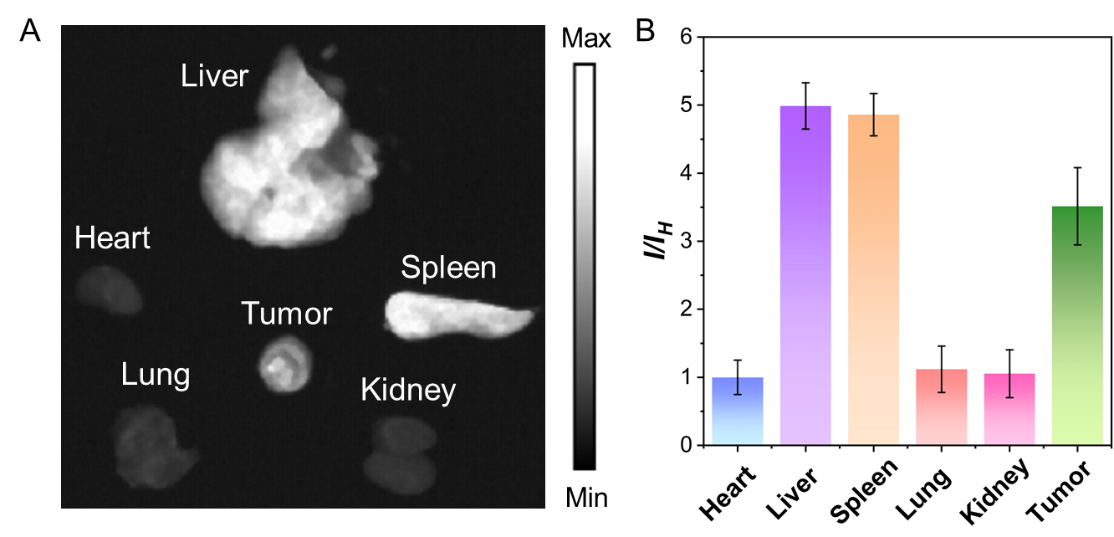


**Figure S47.** (A) The images of the major organs (heart, liver, spleen, lung, kidney) in the NIR-II channel after 24 h post-injection of *b*-3CPFIC NPs. (B) The *I*/*I*_H_ ratio of the major organ (heart, liver, spleen, lung, kidney) after 24 h post-injection of *b*-3CPFIC NPs. *I* represent the PL intensity of the major organ, while *I_H_* indicates the PL intensity of the heart.


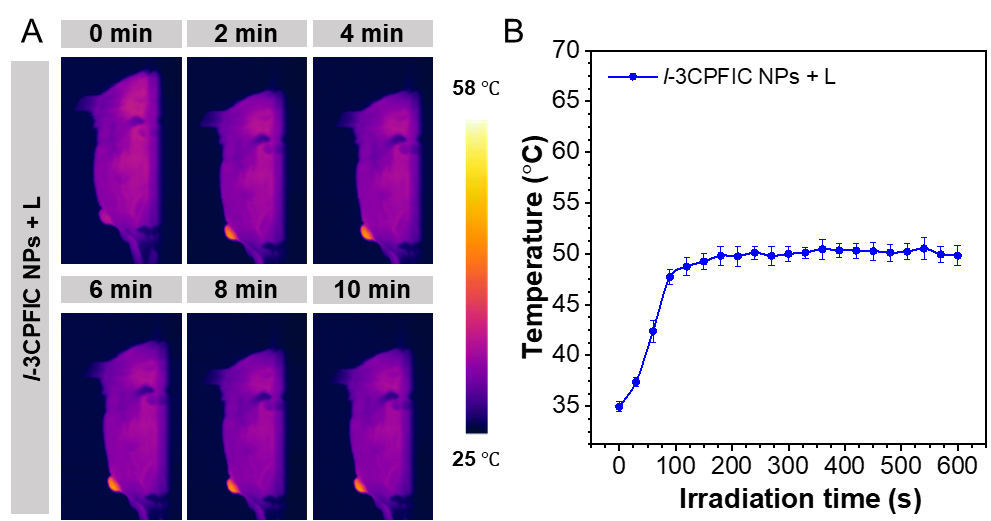


**Figure S48.** (A) The IR thermal imaging of tumor sites after injection of *l*-3CPFIC NPs under laser irradiation (808 nm, 0.8 W cm^-2^) for different times. (B) Corresponding temperature variations of tumor sites in *l*-3CPFIC NPs-treated groups. The data for the PBS control group is shared with Figure 7E and 7F to ensure experimental consistency, as these groups were evaluated simultaneously within the same batch.


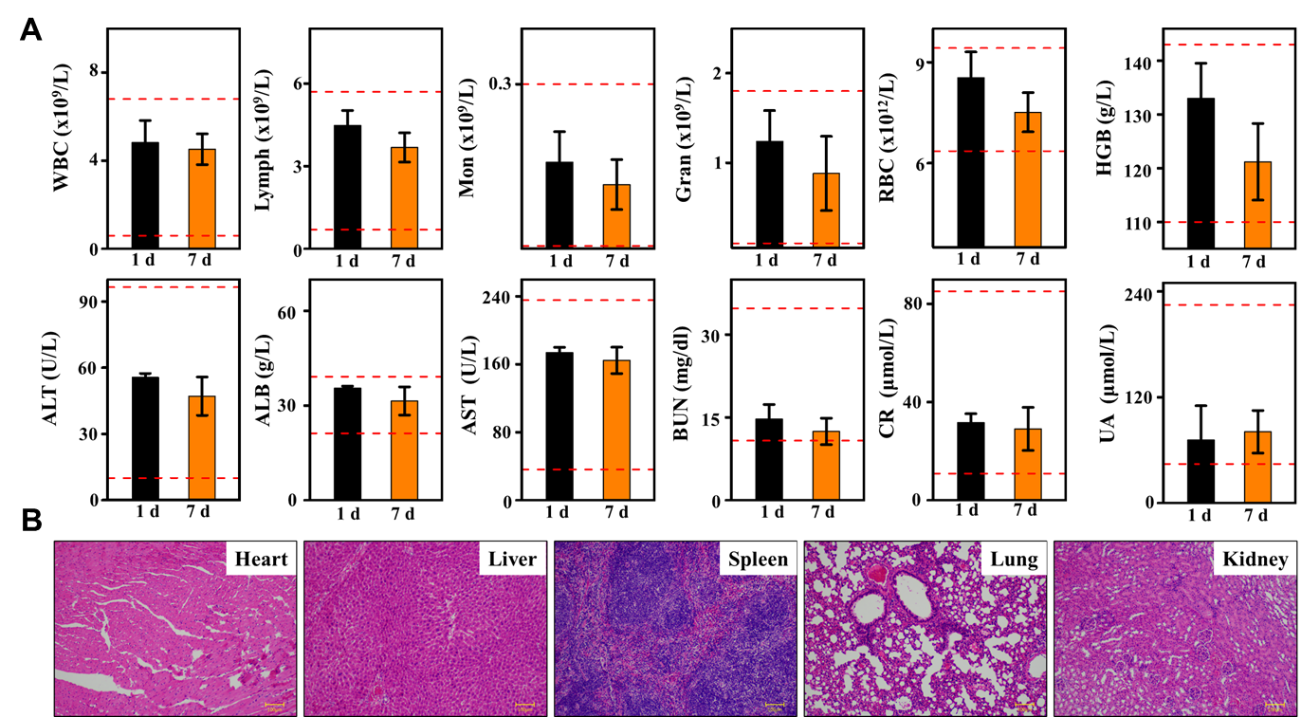


**Figure S49.** (A) Blood biochemistry analysis of the mice receiving therapy. (B) Histological staining (Hematoxylin and Eosin Staining) analysis of major organs collected from the mice of NPs + L group after 16-day treatments.

References

(1) Y. Zhu, H. Lai, H. Guo, D. Peng, L. Han, Y. Gu, Z. Wei, D. Zhao, N. Zheng, D. Hu, L. Xi, F. He, L. Tian, Side-Chain-Tuned Molecular Packing Allows Concurrently Boosted Photoacoustic Imaging and NIR-II Fluorescence. *Angew. Chem. Int. Ed.* **2022**, *61*, e202117433.

(2) H. M. Pan, C. C. Wu, C. Y. Lin, C. S. Hsu, Y. C. Tsai, P. Chowdhury, C. H. Wang, K. H. Chang, C. H. Yang, M. H. Liu, Y. C. Chen, S. P. Su, Y. J. Lee, H. K. Chiang, Y. H. Chan, P. T. Chou, Rational Design of Asymmetric Polymethines to Attain NIR(II) Bioimaging at >1100 nm. *J. Am. Chem. Soc.* **2023**, *145*, 516-526.

(3) H. Wan, J. Yue, S. Zhu, T. Uno, X. Zhang, Q. Yang, K. Yu, G. Hong, J. Wang, L. Li, Z. Ma, H. Gao, Y. Zhong, J. Su, A. L. Antaris, Y. Xia, J. Luo, Y. Liang, H. Dai, A Bright Organic NIR-II Nanofluorophore for Three-Dimensional Imaging into Biological Tissues. *Nat. Commun.* **2018**, *9*, 1171.

(4) H. Shen, F. Sun, X. Zhu, J. Zhang, X. Ou, J. Zhang, C. Xu, H. H. Y. Sung, I. D. Williams, S. Chen, R. T. K. Kwok, J. W. Y. Lam, J. Sun, F. Zhang, B. Z. Tang, Rational Design of NIR-II AIEgens with Ultrahigh Quantum Yields for Photo- and Chemiluminescence Imaging. *J. Am. Chem. Soc.* **2022**, *144*, 15391-15402.

(5) C. Dong, Z. Zhang, H. Wu, X. Liang, S. Pang, K. Wu, J. Sun, X. Dong, L. Sun, X. Gu, C. Zhao, Dual-Modal Imaging-Guided Agent Based on NIR-II Aggregation-Induced Emission Luminogens with Balanced Phototheranostic Performance. *Chem. Sci.* **2024**, *15*, 10969-10979.

(6) Y. Wan, W. Chen, Y. Liu, K. W. Lee, Y. Gao, D. Zhang, Y. Li, Z. Huang, J. Luo, C. S. Lee, S. Li, Neutral Cyanine: Ultra-Stable NIR-II Merocyanines for Highly Efficient Bioimaging and Tumor-Targeted Phototheranostics. *Adv. Mater.* **2024**, *36*, e2405966.

(7) F. Wan, H. Wang, Y. Gu, G. Fan, S. Hou, J. Yu, M. Wang, F. He, L. Tian, Bromine Substitution Improves the Photothermal Performance of pi-Conjugated Phototheranostic Molecules. *Chem. - Eur. J.* **2024**, *30*, e202303502.

(8) Y. Hou, J. Li, G. Jiang, T. Xia, Z. Li, H. Gu, X. Liu, Q. Yao, C. Zhang, W. Liu, J. Du, W. Sun, J. Fan, X. Peng, Synergistic Inter‐ and Intramolecular Aggregation of Dimeric Cyanine Dyes Affords Highly Efficient In Vivo Self‐Delivery and Photothermal Therapy. *Adv. Funct. Mater.* **2024**, *34*, 2316452.

(9) X. Li, F. Fang, B. Sun, C. Yin, J. Tan, Y. Wan, J. Zhang, P. Sun, Q. Fan, P. Wang, S. Li, C. S. Lee, Near-infrared Small Molecule Coupled with Rigidness and Flexibility for High-Performance Multimodal Imaging-Guided Photodynamic and Photothermal Synergistic Therapy. *Nanoscale Horiz.* **2021**, *6*, 177-185.

(10) X. Hu, Q. Jia, Q. Fang, C. Song, R. Zhang, Y. Liang, Z. Yang, J. Wu, H. Li, T. Zhao, D. Zhao, Z. Wang, Synchronously Manipulating the D−A Interaction and Planarity in Semiconducting Polymers to Achieve 84.7% Photothermal Conversion Efficiency for NIR‐II Imaging‐Guided Tumor Therapy. *Adv. Funct. Mater.* **2024**, *34*, 240710.

(11) C. Huang, T. Shi, J. Zhang, Y. Sun, T. Ma, W. Li, Y. Li, H. Qiu, S. Yin, An NIR-II-Absorbing Photothermal Agent Containing Multiple Rotors with Enhanced Photothermal Conversion Capacity for Multimodal-Imaging-Guided Photothermal Therapy. *Dyes and Pigments* **2023**, *210*, 110932.

(12) B. Lu, H. Quan, Z. Zhang, T. Li, J. Wang, Y. Ding, Y. Wang, X. Zhan, Y. Yao, End Group Nonplanarization Enhances Phototherapy Efficacy of A-D-A Fused-Ring Photosensitizer for Tumor Phototherapy. *Nano Letters* **2023**, *23*, 2831-2838.

(13) K. X. Teng, L. Y. Niu, Q. Z. Yang, Supramolecular Photosensitizer Enables Oxygen-Independent Generation of Hydroxyl Radicals for Photodynamic Therapy. *J. Am. Chem. Soc.* **2023**, *145*, 4081-4087.

(14) L. Li, C. Shao, T. Liu, Z. Chao, H. Chen, F. Xiao, H. He, Z. Wei, Y. Zhu, H. Wang, X. Zhang, Y. Wen, B. Yang, F. He, L. Tian, An NIR-II-Emissive Photosensitizer for Hypoxia-Tolerant Photodynamic Theranostics. *Adv. Mater.* **2020,** *32*, e2003471.

(15) D. Van Der Spoel, E. Lindahl, B. Hess, G. Groenhof, A. E. Mark, H. J. C. Berendsen, "GROMACS 2015: A High-Performance Software Package for Molecular Simulation." SoftwareX, **2015,** *2*, 19-25.

(16) M. J. Frisch, G. W. Trucks, H. B. Schlegel, G. E. Scuseria, M. A. Robb, J. R. Cheeseman, G. Scalmani, V. Barone, G. A. Petersson, H. Nakatsuji, X. Li, M. Caricato, A. V. Marenich, J. Bloino, B. G. Janesko, R. Gomperts, B. Mennucci, H. P. Hratchian, J. V. Ortiz, A. F. Izmaylov, J. L. Sonnenberg, Williams, F. Ding, F. Lipparini, F. Egidi, J. Goings, B. Peng, A. Petrone, T. Henderson, D. Ranasinghe, V. G. Zakrzewski, J. Gao, N. Rega, G. Zheng, W. Liang, M. Hada, M. Ehara, K. Toyota, R. Fukuda, J. Hasegawa, M. Ishida, T. Nakajima, Y. Honda, O. Kitao, H. Nakai, T. Vreven, K. Throssell, J. A. Montgomery, J. E. Peralta, F. Ogliaro, M. J. Bearpark, E. N. Brothers, K. N. Kudin, V. N. Staroverov, T. A. Keith, R. Kobayashi, J. Normand, K. Raghavachari, A. P. Rendell, J. C. Burant, S. S. Iyengar, J. Tomasi, M. Cossi, J. M. Millam, M. Klene, C. Adamo, R. Cammi, J. W. Ochterski, R. L. Martin, K. Morokuma, O. Farkas, J. B. Foresman, D. J. Fox, *Gaussian 16 Rev. C.01*, Wallingford, CT, **2016**.

(17) W. Humphrey, A. Dalke, K. Schulten, VMD: Visual Molecular Dynamics. *J. Mol. Graphics* **1996,** *14*, 33-38.

(18) T. Lu, F. Chen, Multiwfn: A Multifunctional Wavefunction Analyzer. *J. Comput. Chem.* **2012,** *33*, 580-592.

(19) T. Lu, A Comprehensive Electron Wavefunction Analysis Toolbox for Chemists, Multiwfn. *J. Chem. Phys.* **2024,** *161*, 082503.

(20) C. Lefebvre, G. Rubez, H. Khartabil, J.-C. Boisson, J. Contreras-García, E. Hénon, Accurately Extracting the Signature of Intermolecular Interactions Present in the NCI Plot of the Reduced Density Gradient versus Electron Density. *Phys. Chem. Chem. Phys.* **2017**, *19*, 17928-17936.

(21) J. C. Cacaccio, F. A. Durrani, J. R. Missert, R. K. Pandey, Photodynamic Therapy in Combination with Doxorubicin Is Superior to Monotherapy for the Treatment of Lung Cancer. *Biomedicines***2022***, 10,* 857.

(22) E. Demiderenko, T. W. Miller, Statistical determination of synergy based on Bliss definition of drugs independence, *PLoS One* **2019**, *14*, e0224137.

(23) B. Li, Y. Gan, K. Yang, E. Pang, X. Ren, S. Zhao, D. He, F. Zhao, B. Wang, P. Yin, X. Song, M. Lan, Acceptor-Donor-Acceptor Structured Phototheranostics for Near-Infrared II Fluorescent and Photoacoustic Imaging-Guided Photodynamic and Photothermal Synergistic Therapy. *Sci. China Mater.* **2022**, *66*, 385-394.

(24) L. Yuan, S. Liang, C. Xiao, Q. Chen, W. Li, Near-Infrared Nonfullerene Acceptors Based on 4H-Cyclopenta[1,2-b:5,4-b']dithiophene for Organic Solar Cells and Organic Field-Effect Transistors. *Chem. Asian J.* **2021**, *16*, 4171-4178.
